# TCR γδ cell–specific STAT5 gain of function induces a druggable chronic human immune dysregulation

**DOI:** 10.70962/jhi.20250175

**Published:** 2026-08-10

**Authors:** Benedikt J. Meyer, José Pedro Loureiro, Vladimir Nosi, Robin Hupfer, Adhideb Ghosh, Fabio Poletti, Annaïse Jauch, Julia Hirsiger, Caroline Berkemeier, Ingmar Heijnen, Jan Dirks, Ilaria Alborelli, Thomas Menter, Alexandar Tzankov, Christoph Hess, Alexander A. Navarini, Christoph T. Berger, Lucia Mori, Gennaro De Libero, Mike Recher

**Affiliations:** 1 Immunodeficiency Laboratory, Department of Biomedicine, University of Basel, Basel, Switzerland; 2 Experimental Immunology Laboratory, Department of Biomedicine, University of Basel and University Hospital, Basel, Switzerland; 3 Functional Genomics Center Zurich, ETH Zurich, and University of Zurich, Zurich, Switzerland; 4 Translational Immunology Laboratory, Department of Biomedicine, University of Basel, Basel, Switzerland; 5 Laboratory Medicine, Division Medical Immunology, University Hospital Basel, Basel, Switzerland; 6 Laboratory Medicine, Division Diagnostic Hematology, University Hospital Basel, Basel, Switzerland; 7 Pathology, Institute of Medical Genetics & Pathology, University Hospital Basel, Basel, Switzerland; 8 Immunobiology Laboratory, Department of Biomedicine, University of Basel, Basel, Switzerland; 9 Department of Medicine, Cambridge Institute of Therapeutic Immunology & Infectious Disease, University of Cambridge, Cambridge, UK; 10 Dermatology and Skin Laboratory, Department of Biomedicine Basel, University Hospital Basel, Basel, Switzerland; 11 University Center for Immunology, University Hospital Basel, Basel, Switzerland

## Abstract

Within a prospective cohort of patients with immune dysregulation, we identified several individuals with chronically increased proportions of TCR γδ cells but normal peripheral lymphocyte counts. Among those, we identified one individual with a TCR γδ cell–specific heterozygous p.Y665F STAT5B gain-of-function mutation. Recurrent oral aphthous lesions, susceptibility to infection, arthralgia, and fatigue, were linked to relatively elevated numbers of γδ T cells expressing a Vγ9Vδ2 TCR, displaying hyperphosphorylation of STAT5 upon in vitro IL-2 stimulation. The TCR Vγ9Vδ2 cells exhibited enhanced proliferative response to (E)-4-hydroxy-3-methyl-but-2-enyl pyrophosphate and dysregulated cytokine production. The TCR γδ cell transcriptome revealed the suppression of the default Th17 program, along with inhibition of *RORC* and *MAF* expression. The JAK inhibitor baricitinib improved clinical features of the observed immune dysregulation and reduced the frequency of peripheral TCR Vγ9Vδ2 cells. Thus, functionally altered TCR γδ cells may underlie chronic immune dysregulation of unknown molecular cause, demonstrated here to be amenable to tailored immune modulation.

## Teaser

A subset of patients evaluated for immune dysregulation presents with increased proportions of TCR γδ cells despite normal total lymphocyte counts. Corresponding to this phenotype, the authors report a case of a somatic TCR γδ–specific STAT5B mutation that is molecularly amenable to tailored immune modulation. This raises awareness of druggable TCR γδ–intrinsic, genetically determined immune dysregulation.

## Introduction

Susceptibility to infection is orchestrated by the host’s immune system. Besides numerous secondary causes of immunodeficiency (1), inborn errors of immunity (IEI) are a rapidly growing group of immune dysregulation entities due to germline mutations in nonredundant immune system genes (2). In addition, somatic mutations in the same genes may lead to phenocopies of IEI (3). IEI are still not diagnosed in many patients, or diagnosis is delayed for many years (4).

Signal transducer and activator of transcription 5 (STAT5) proteins play a key role in regulating hematopoiesis and hepatocyte function (5). STAT5A and STAT5B transcription factors form homo- or heterodimeric complexes that regulate differential gene expression (5, 6). The STAT5 dimerization is initiated upon binding of cytokines such as interleukin-2 (IL-2) or growth factors to their respective receptors on the cell surface. This process triggers the signaling via JAK1, JAK2, and JAK3 tyrosine kinases, leading to the nuclear translocation and transcriptional activities of STAT5 (5, 7).

STAT5 insufficiency resulting from germline biallelic loss-of-function mutations or dominant-negative monoallelic mutations can cause eczema, autoimmunity, and growth hormone–insensitive growth failure (8, 9). A germline STAT5 gain-of-function (GOF) mutation has been identified in two patients with atopic disease (10). In addition, somatic GOF mutations causing STAT5 overactivity drive inflammation, autoimmunity, myeloid, or lymphoproliferative diseases, and promote cancer, depending on the mutated cell lineage (5, 11, 12, 13). In malignant cells, persistent activation of STAT5 facilitates evasion of cell cycle control, playing a crucial role in leukemogenesis and the proliferation of various tumors (14).

T lymphocytes can express TCRs that result from pairing either α and β chains (TCR αβ) or γ and δ chains (TCR γδ). In humans, TCR γδ cells constitute 0.5–16% of the total circulating T cells and are present at higher frequencies in peripheral tissues (15). The TCRs γδ can recognize a broad range of molecules, including various members of the butyrophilin family (16). Most circulating TCR γδ cells in humans express a TCR Vγ9Vδ2 heterodimer and are activated by phosphorylated metabolites shared among several bacteria (17). The widespread distribution of bacterial phosphoantigens (pAgs) leads to continuous activation and supports their expansion. This constant stimulation induces TCR Vγ9Vδ2 cells to mature into effector cells that are ready to respond upon the subsequent antigen encounter. Consequently, these T cells are considered intermediate protagonists of both innate and adaptive immunity (18).

Dysregulation of TCR γδ cells contributes to the pathogenesis of systemic sclerosis, psoriasis, rheumatoid arthritis, and systemic lupus erythematosus, among others (18, 19, 20). Upon activation, they more frequently display T helper 1 (Th1) cytokine profiles, but may also rarely exhibit Th2 or Th17 profiles, secreting either IFN-γ, TNF-α, and IL-2; IL-4 and IL-13; or IL-17, IL-21, and IL-23, respectively (21, 22, 23). These pleiotropic functions contribute to their participation in diverse immune responses.

Both TCR αβ and TCR γδ T cells may appear as T cell large granular lymphocytes (T-LGL) in peripheral blood, with an activated morphology on blood smear microscopy and flow cytometric expression of typical natural killer (NK) cell markers such as CD16, CD56, and/or CD57. T-LGL are not pathologic per se, and increased numbers (reactive expansions) of T-LGL are observed during infections or inflammation (24). In contrast, T-LGL leukemia is referred to as a clonal, distinct (typically >2 x 10^9^/L) expansion of T-LGL that may be complicated by cytopenias, lymphoproliferation, and/or autoimmune diseases (24).

Here, we describe several patients with chronic immune dysregulation, normal lymphocyte counts, but chronically elevated TCR γδ vs. TCR αβ ratios. In one of the patients, molecular analysis revealed a TCR γδ–specific STAT5 GOF mutation as the molecular driver. Tailored treatment with the JAK inhibitor baricitinib successfully normalized hyperinflammation and reduced peripheral TCR γδ cells.

## Results

### Identification of patients with elevated TCR γδ cells as the main abnormality in a prospective multicenter immune dysregulation cohort

We screened our prospective multicenter adult immune dysregulation cohort, currently comprising >600 patients. We identified five individuals with normal absolute lymphocyte counts who exhibited a reduced TCR αβ/TCR γδ ratio as the primary immunophenotypic abnormality (Table 1). All were adults with chronic susceptibility to airway infections, mainly chronic sinusitis. None of the patients displayed antibody deficiency. In all patients, the increased proportion of TCR γδ cells and the normal total lymphocyte counts remained stable over time (most had several years of follow-up) (Table 1). The TCR γδ cells were classified as T-LGL by the diagnostic hematology flow cytometric assessment, based on variable expression of the NK cell markers CD16, CD56, and CD57 (Table 1). Next-generation sequencing (NGS)–based TCR sequencing was performed in four patients, revealing that >85% of the TCR γδ cell expansion was polyclonal in all (Table 1). In the remaining patient, PCR-based testing showed biclonal TCR expansion over a polyclonal background (Table 1). The goal of this study was to characterize potential molecular culprits driving the predominant polyclonal relative increase in TCR γδ cells in these patients and to study TCR γδ cell function.

**Table 1.** Clinical, immunophenotypic, and molecular characteristics of five normolymphocytic patients with elevated TCR γδ frequencies

FuGe cohort number	44 (P1)		86		528		558		581	
Year of birth	1986		1985		1989		1998		1961	
Clinical phenotype, associated atopy	Susceptibility to airway infections; oral erosions; elevated IgE		Childhood-onset allergic asthma; recurrent viral-like airway infections; house dust mite desensitization; normal IgE		Recurrent viral-like airway infections (20× per year); tonsillectomy; low IgE		Recurrent tonsillitis and sinusitis since childhood; splenectomy due to spherocytosis; normal IgE		Chronic rhinosinusitis without polyps; asthma; ENT surgery; low IgE	
Evaluation date	2017	2025	2019	2024	2023	2024	2024	2024	2024	2025
Neutrophils absolute (G/L); Ref: 1.3–6.7	6.02	4.61	5.94	3.82	6.29	5.43	3.51	N.D	2.87	5.5
Lymphocytes absolute (G/L); Ref: 0.9–3.3	2.39	1.72	**3.74**	2.51	2.82	2.2	1.91	N.D	2.03	1.99
Lymphocytes (% of leukocytes); Ref: 19–48	26.23	24.20	35.10	35.20	28.40	26.50	30.80	N.D	25.60	24.70
Absolute T cell count (cells/μl); Ref: 742–2,750	1,857	1,317	**3,132**	2,274	2,629	1,947	1,730	1,792	1,728	1,830
Absolute γδ T cell count (cells/μl)	612	233	1,221	932	565	368	847	914	373	389
γδ T cells (% of total T cells); Ref: 0.5–16	**33**	**17.7**	**39**	**41**	**21.5**	**18.9**	**49**	**51**	**21.6**	**21.3**
T cell clonality (method)	6.7% (NGS)	13% (NGS)	Biclonal (multiplex PCR)	N.D	7% (NGS)	N.D	N.D	12.7% (NGS)	14% (NGS)	N.D
γδ T-LGL (% of T cells) as assessed by diagnostic hemato-immunologic flow cytometry	25%	12%	42%	N.D	N.D	17%	N.D	40%	25%	N.D
γδ T cell flow phenotype	CD5dim, CD2^+^, CD7^+^, CD52^+^, CD30^−^, CD16^+^, CD56^+^, CD57^−^	CD5dim, CD2^+^, CD7^+^, CD52^+^, CD30^−^, CD16^+^, CD56^+^, CD57^−^	CD5dim, CD2^+^, CD7^+^, CD52^+^, CD30^−^, CD16^−^, CD56^−^, CD57^+^	N.D	N.D	CD5dim, CD2^+^, CD7^+^, CD16^+^, CD56^+^, CD57^+^	N.D	CD5dim, CD2^+^, CD7^+^, CD16^+^, CD56^+^, CD57^+^	CD5dim, CD2^+^, CD7^+^, CD52^+^, CD30^−^, CD16^+^, CD56^+^, CD57^+^	N.D
STAT5B mutation (method)	Yes (NGS-sorted γδ T cells)	N.D	No (NGS-sorted γδ T cells)	N.D	N.D	No (Sanger sequencing; PBMCs)	N.D	No (Sanger sequencing; PBMCs)	No (Sanger sequencing; PBMCs)	N.D
STAT3 mutation (method)	No (NGS-sorted γδ T cells)	N.D	No (NGS-sorted γδ T cells)	N.D	N.D	N.D	N.D	N.D	N.D	N.D
Treatment	Baricitinib		Watch and wait		Watch and wait		Watch and wait		Watch and wait	

N.D, not done; Ref, reference range. Values above reference range are indicated in bold. Sanger sequencing of bulk PBMC may fail to detect low-variant-allele-frequency mutations in both STAT3 and STAT5B.

### Detailed clinical and immunologic characteristics of the index patient (patient 1 [P1])

**Figure 1.**
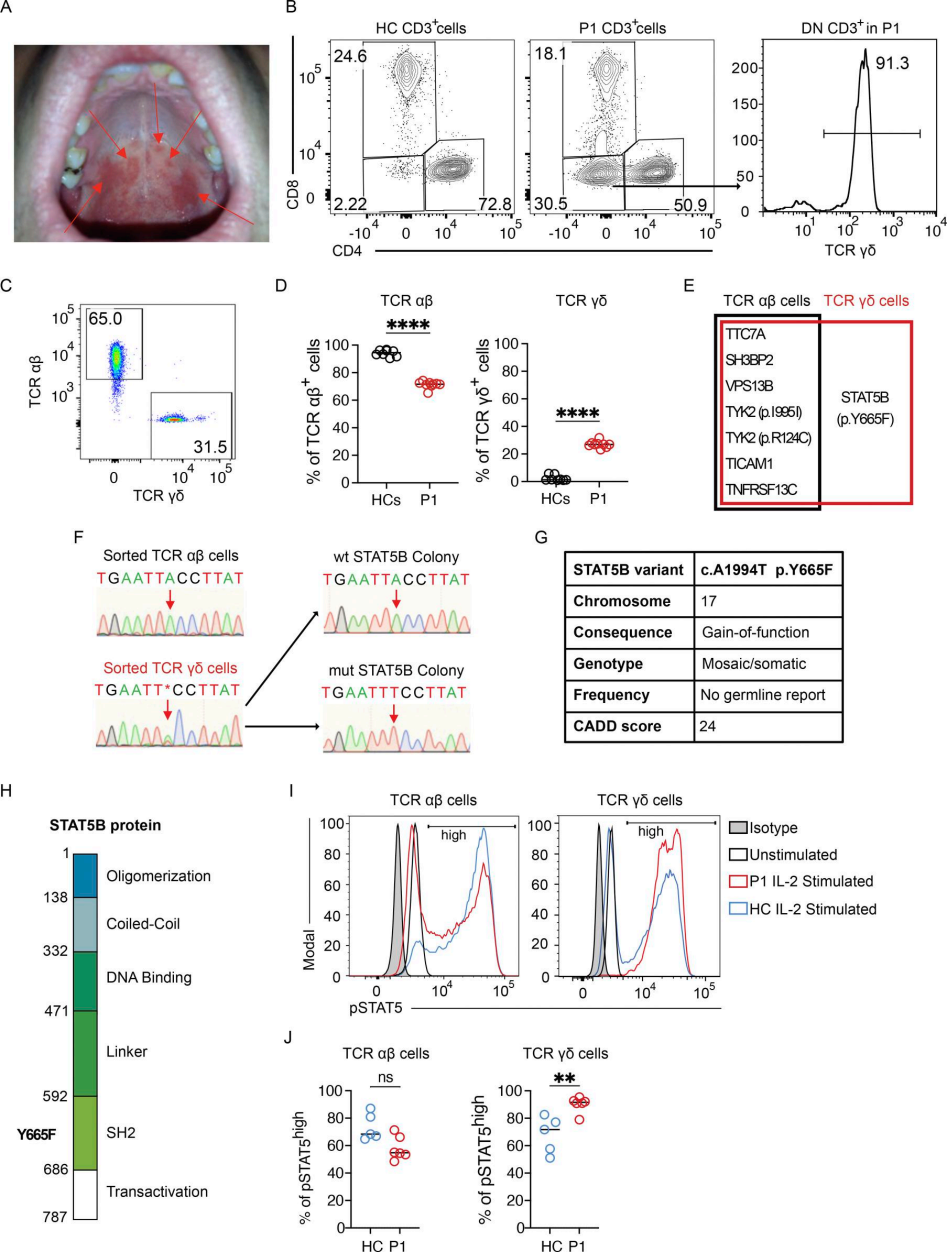
**Identification of a STAT5 GOF mutation selectively in TCR γδ cells of P1. (A)** Representative picture of the oral mucosa from P1 at diagnosis. The picture shows erosive stomatitis (red arrows). **(B)** Representative flow cytometry plots of CD4 and CD8 expression on total T cells from a HC and P1, followed by TCR γδ expression on CD4^−^CD8 DN cells. **(C)** Representative flow cytometry plots of TCR γδ and TCR αβ cells within live CD3^+^ PBMCs from P1. **(D)** Summary of T cells from HCs vs. P1 expressing TCR αβ (left) or TCR γδ (right). The analyzed samples from P1 were collected throughout 14 years; each dot corresponds to an independent experiment. Scatter plots of percentages and the median of seven independent measurements. Mann–Whitney test. ****P < 0.0001. **(E)** List of PID gene variants detected by WES from the genomic DNA of freshly sorted TCR αβ and TCR γδ cells. Found PID variants were filtered for CADD scores >15 and allele frequencies <0.05. The p.Y665F STAT5B missense variant was the only one found exclusively in TCR γδ cells. **(F)** Sanger sequencing of p.Y665F STAT5B region amplified from cDNA of freshly sorted TCR αβ (top panel) and TCR γδ (bottom panel) cells. A STAT5B-specific PCR product amplified from cDNA containing the region of the p.Y665F mutation was cloned into a plasmid and sequenced. In total, 23 colonies containing plasmids with the PCR product were sequenced and five colonies carried the STAT5B mutation (21%). Sanger sequencing results are shown from a colony containing wild-type STAT5B (top right panel) and a colony containing mutant STAT5B (bottom right panel). The data correspond to one experiment. **(G)** Overview for the p.Y665F (c.A1994T) STAT5B variant. **(H)** Scheme of STAT5B protein with the location of the p.Y665F STAT5B variant in the SH2 domain of STAT5B. **(I)** Representative histogram of pSTAT5 expression in TCR αβ (top) or TCR γδ (bottom) cells from HCs vs. P1 rested overnight and stimulated or not with IL-2 for 30 min. The matched isotype is shown in black. **(J)** Summary of percentages of pSTAT5 in TCR αβ or TCR γδ cells from HCs and P1 rested overnight and stimulated with IL-2 for 30 min. Mann–Whitney test. **P < 0.01. Each dot corresponds to an independent experiment. DN, double negative; PID, primary immunodeficiency.

**Figure S1.**
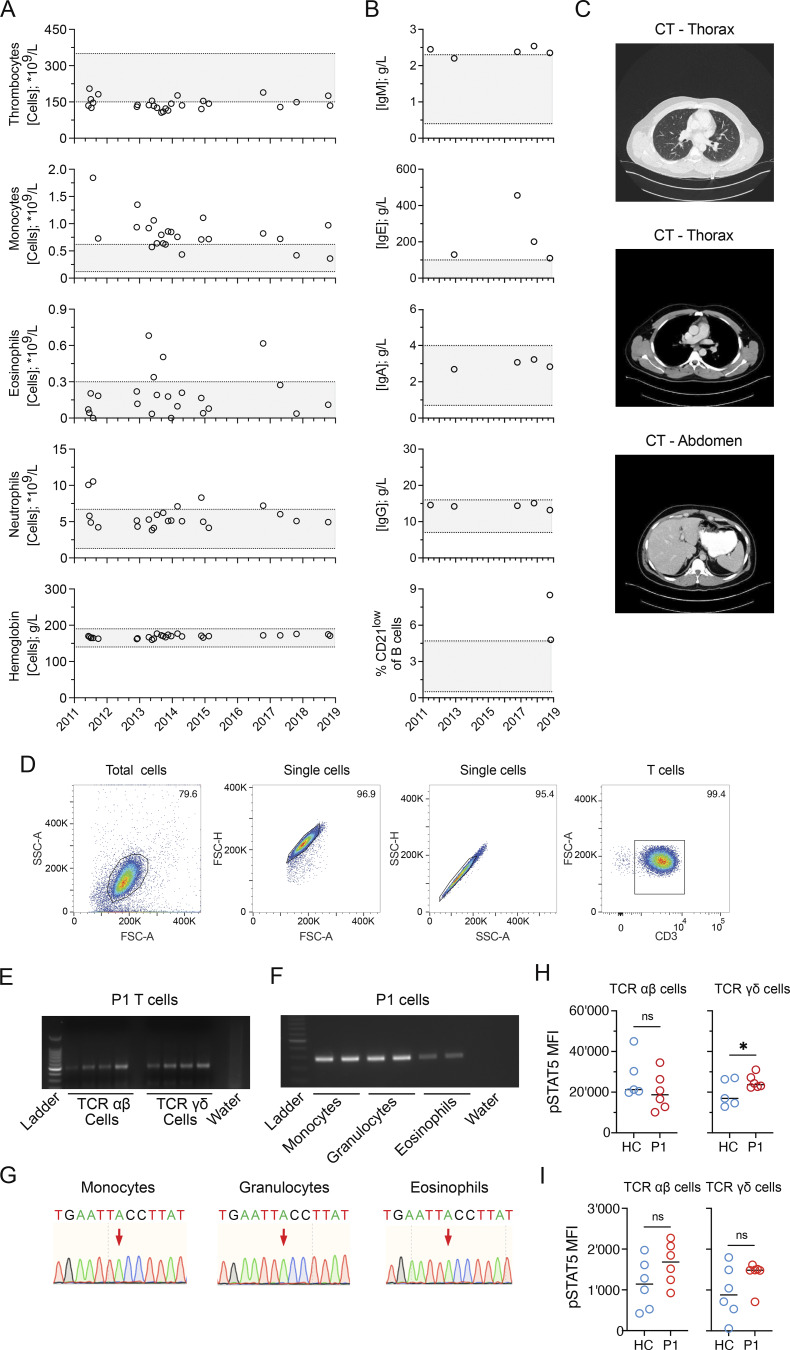
**Longitudinal blood and immune cell subpopulation counts in P1 prior to JAK inhibition therapy, CT scan, and screening for STAT5B GOF mutation in monocytes, granulocytes, and eosinophils. (A)** Levels of thrombocytes, monocytes, eosinophils, neutrophils, and hemoglobin from hemograms performed between 2011 and 2019. Gray areas delineate the normal reference values for each parameter. **(B)** Immunoglobulin levels in different hemograms performed between 2011 and 2019. Gray areas delineate the normal reference values for each parameter. **(C)** CT scan of the thorax and abdomen performed in 2013. **(D)** Gating strategy to assess TCR, CD4, CD8, and pSTAT5 of T cells, which were isolated from PBMCs. Lymphocytes were sequentially gated according to FSC-A/SSC-A, followed by singlets (FSC-A/FSC-H and SSC-A/SSC-H). T cells were then gated as CD3^+^ cells. **(E and F)** Agarose gel electrophoresis of PCR product amplifying the p.Y665F STAT5B missense variant from cDNA of monocytes, granulocytes, and eosinophils. **(E)** Lane 1—ladder with size marker; lanes 2, 3, 4, 5—TCR αβ cells with different cDNA input; lanes 7, 8, 9, 10—TCR γδ cells with different amounts of cDNA input from the same P1-derived sample; lane 12—water. **(F)** Lane 1—ladder with size marker; lanes 2, 3—monocytes; lanes 4, 5—granulocytes; lanes 6, 7—eosinophils; lane 8—water; duplicates correspond to different cDNA amount inputs from the same P1-derived sample. **(G)** Sanger sequencing of p.Y665F STAT5B region amplified from cDNA of PBMC-derived freshly sorted monocytes (left), granulocytes (middle), and eosinophils (right). The data correspond to one experiment. **(H and I)** Summary of pSTAT5 MFI in TCR αβ or TCR γδ cells from five different HCs and P1 from five different time points. Cells were rested overnight and incubated in the presence (H) or absence (I) of IL-2. Unpaired *t* test. *P < 0.05. Every dot corresponds to an independent experiment. CT, computed tomography. Source data are available for this figure: SourceData FS1.

P1 is a Caucasian male who has been monitored at our hospital for >16 years, starting at age 24. At his first visit, P1 presented with recurrent, large, and multiple oral erosive lesions that occurred twice monthly since young adulthood (Fig. 1 A), accompanied by chronic arthralgia and fatigue. P1 reported a susceptibility to prolonged, mostly mild (viral) upper respiratory tract infections that typically did not require antibiotic treatment. Family history for similar manifestations was negative. P1 repeatedly showed mild, inconsistent thrombocytopenia, monocytosis, and variable neutrophilia and eosinophilia (Fig. S1 A). Serum IgG and IgA were within the reference range, while IgE (456 IU/ml) and IgM (2.54 g/L, polyclonal) were slightly elevated (Fig. S1 B). Hemoglobin levels remained normal at all collection times, as was the C-reactive protein (except in acute infections, where it was mildly elevated). Repeated sonography and computed tomography (CT)–based imaging showed neither lymphadenopathy nor splenomegaly (Fig. S1 C). Additional immunologic abnormalities included increased positive antinuclear autoantibodies with a nuclear dot immunofluorescence staining pattern, and elevated CD21^low^ B cells (Fig. S1 B). The latter may indicate hyperinflammation driven by IFN-γ (25). The patient never met diagnostic criteria for systemic lupus erythematosus. Pathergy skin testing and HLA-B51 tests were negative, arguing against Behcet’s syndrome. A bone marrow biopsy revealed normal erythro- and myelopoiesis, along with reactive interstitial lymphocytic infiltration, without signs of lymphoma. Flow cytometry analysis of peripheral blood mononuclear cells (PBMCs) confirmed the overrepresentation of CD4^−^CD8^−^ T cells, comprising ∼30% of all CD3^+^ T cells (Fig. 1 B and Fig. S1 D). Most of these T cells expressed TCR γδ (Fig. 1 B). The relative increase in TCR γδ vs. TCR αβ cells remained stable over the following 9 years in the absence of immune-modulating therapy (Fig. 1, C and D). The frequencies of NK cells and the different TCR αβ cell subpopulations (naive, central memory, effector memory, CD4 regulatory T [Tregs], CD4 Tfh) were within normal ranges, except for Tregs, which were slightly relatively increased (Table 2). NGS-based TCRγ sequencing revealed seven different clones with >1% frequency, the top clone accounting for 6.7% (Table 1 and Table 3). Thus, the majority of the relative TCR γδ expansion was polyclonal.

**Table 2.** Peripheral blood–derived lymphocyte subsets of P1

​	Reference range	10/01/18	10/29/18	01/19	04/19	09/19	03/20	01/21
T cells absolute (cells/μl)	742–2,750	1,688	1,699	1,595	1,581	1,429	1,508	1,569
T cells relative (% of lymphocytes)	55–86	72	73	75	75	72	72	67
CD4 T cells absolute (cells/μl)	404–1,612	779	972	881	835	751	845	806
CD4 T cells relative (% of lymphocytes)	33–58	34	35	41	41	39	40	35
Naive CD4 (% of CD4)	15.7–54.7	–	–	–	30.6	35.7	31.1	–
Central memory CD4 (% of CD4)	8–28.9	–	–	–	24.9	20.4	26.8	–
Effector memory CD4 (% of CD4)	16.8–57.4	–	–	–	39.9	38.2	38.5	–
TEMRA CD4 (% of CD4)	3.6–23.2	–	–	–	4.6	5.7	3.6	–
RTE CD4 (% of naive CD4)	14.1–37.2	–	–	–	26.2	30.9	27	–
Treg (% of CD4)	6.1–11	–	–	–	**11.9**	**11.9**	10.2	–
TFH (% of CD4)	6.9–19.1	–	–	–	15	12.7	14.5	–
Activated CD4 (% of CD4)	4.1–15.6	–	–	–	7.4	5.9	5.8	–
CD8 T cells absolute (cells/μl)	220–1,129	720	879	678	620	472	459	560
CD8 T cells relative (% of lymphocytes)	11–39	31	32	32	30	24	22	25
Naive CD8 (% of CD8)	7–62.5	–	–	–	44.4	45.9	46.5	–
Central memory CD8 (% of CD8)	0.6–4.4	–	–	–	2.5	1.1	**4.5**	–
Effector memory CD8 (% of CD8)	4.3–64.5	–	–	–	31.8	32	34.1	–
TEMRA CD8 (% of CD8)	8.1–60.5	–	–	–	21.3	20.9	14.9	–
Activated CD8 (% of CD8)	8.7–45.2	–	–	–	*7.7*	*7.1*	*4.4*	–
CD4/CD8 double-negative TCR αβ cells (% of T cells)	<2.5%	–	–	–	1.4	1.6	1.6	–
B cells absolute (cells/μl)	80–616	334	424	312	262	376	416	485
B cells relative (% of lymphocytes)	5–22	14	15	15	12	19	20	20
NK cells absolute (cells/μl)	84–724	314	276	217	258	184	156	263
NK cells relative (% of lymphocytes)	5–26	13	10	10	12	9	8	11

RTE, recent thymic emigrants; TEMRA, T effector memory cells with CD45RA expression; TFH, T follicular helper cells; Treg, regulatory T cells; activated, MHCII+. Values above reference range are indicated in bold, and values below reference range in italics.

**Table 3.** NGS-based TCRγ clones in P1 pre-JAK inhibition (2017) vs. under JAK inhibition (2024)

*TRGV* gene	***TRGJ*** gene	CDR3γ amino acid code	CDR3γ nucleotide code	Frequency (2017)	Frequency (2024)
*TRGV9*	*TRGJP*	ALWEVRELGKKIKV	5′-GCC​TTG​TGG​GAG​GTG​CGG​GAG​TTG​GGC​AAA​AAA​ATC​AAG​GTA-3′	0.067	0.133
*TRGV9*	*TRGJ1*	ARPIYRWET	5′-GCC​CGC​CCC​ATA​TAT​CGG​TGG​GAA​ACT​C-3′	0.030	0.017
*TRGV10*	*TRGJ1*	AAWVPKGET	5′-GCT​GCG​TGG​GTC​CCA​AAG​GGA​GAA​ACT​C-3′	0.023	0.014
*TRGV9*	*TRGJ1*	ASGVR*L	5′-GCC​TCG​GGA​GTG​CGT​TGA​CTC-3′	0.015	<0.002
*TRGV10*	*TRGJ1*	AAWDYWET	5′-GCT​GCG​TGG​GAT​TAC​TGG​GAA​ACT​C-3′	0.014	0.007
*TRGV10*	*TRGJ1*	AAWDLRN	5′-GCT​GCG​TGG​GAT​TTA​AGA​AAC​TC-3′	0.013	<0.002
*TRGV10*	*TRGJ1*	AAWGELL*ET	5′-GCT​GCG​TGG​GGA​GAA​TTA​TTA​TAA​GAA​ACT​C-3′	0.013	0.047
*TRGV9*	*TRGJ1*	ALWEVEET	5′-GCC​TTG​TGG​GAG​GTG​GAA​GAA​ACT​C-3′	0.009	<0.002
*TRGV10*	*TRGJ1*	AAWET	5′-GCT​GCG​TGG​GAA​ACT​C-3′	0.008	<0.002
*TRGV9*	*TRGJ1*	ALWEERN	5′-GCC​TTG​TGG​GAG​GAA​AGA​AAC​TC-3′	0.008	<0.002
*TRGV9*	*TRGJ1*	ALWEARWKL	5′-GCC​TTG​TGG​GAG​GCG​AGG​TGG​AAA​CTC-3′	0.008	<0.002
*TRGV9*	*TRGJ1*	ALWEVPPREL	5′-GCC​TTG​TGG​GAG​GTC​CCC​CCT​AGG​GAA​CTC-3′	0.008	<0.002
*TRGV9*	*TRGJ1*	ALWTQEET	5′-GCC​TTG​TGG​ACG​CAG​GAA​GAA​ACT​C-3′	0.006	<0.002
*TRGV9*	*TRGJ1*	ALWEITILRS	5′-GCC​TTG​TGG​GAG​ATT​ACG​ATT​TTA​AGA​AGC​TC-3′	0.006	<0.002
*TRGV9*	*TRGJ1*	ALWEVVIIRN	5′-GCC​TTG​TGG​GAG​GTG​GTT​ATT​ATA​AGA​AAC​TC-3′	0.006	<0.002
*TRGV9*	*TRGJ1*	ALWEVRL*ET	5′-GCC​TTG​TGG​GAG​GTG​CGG​GGT​TAT​AAG​AAA​CTC-3′	0.006	<0.002
*TRGV9*	*TRGJ1*	ALWEVL*ET	5′-GCC​TTG​TGG​GAG​GTA​TTA​TAA​GAA​ACT​C-3′	0.006	<0.002
*TRGV9*	*TRGJP*	ALWEVQELGKKIKV	5′-GCC​TTG​TGG​GAG​GTG​CAA​GAG​TTG​GGC​AAA​AAA​ATC​AAG​GTA-3′	0.005	<0.002
*TRGV9*	*TRGJ1*	ALWDTIRN	5′-GCC​TTG​TGG​GAT​ACT​ATA​AGA​AAC​TC-3′	0.005	<0.002
*TRGV10*	*TRGJ1*	AAWDPGN	5′-GCT​GCG​TGG​GAT​CCG​GGA​AAC​TC-3′	0.004	<0.002
*TRGV9*	*TRGJ1*	ALWEVRET	5′-GCC​TTG​TGG​GAG​GTG​CGG​GAA​ACT​C-3′	0.004	<0.002
*TRGV9*	*TRGJ1*	ALWEVKAIRN	5′-GCC​TTG​TGG​GAG​GTG​AAA​GCC​ATA​AGA​AAC​TC-3′	0.004	<0.002
*TRGV9*	*TRGJ1*	ALWEVGYKKL	5′-GCC​TTG​TGG​GAG​GTG​GGT​TAT​AAG​AAA​CTC-3′	0.004	<0.002
*TRGV10*	*TRGJ1*	AAWDYKKL	5′-GCT​GCG​TGG​GAT​TAT​AAG​AAA​CTC-3′	0.003	<0.002
*TRGV4*	*TRGJ1*	ATLGWT	5′-GCC​ACC​CTC​GGT​TGG​ACT​C-3′	0.003	<0.002

### Detection of a TCR γδ cell–intrinsic GOF mutation in *STAT5B* in P1

To investigate a possible genetic predisposition for the preferential presence of TCR γδ cells, we performed whole-exome sequencing (WES) of P1’s flow cytometry–sorted αβ vs. γδ T cell subpopulations. We filtered the WES results for rare immune system gene variants with low allele frequency (<0.01) and high combined annotation-dependent depletion (CADD) score (>15), revealing a c.A1994T; p.Y665F STAT5B variant present in TCR γδ cells only (Fig. 1, E and F; and Table 4), in 14 out of 68 reads (21%). This suggested that the *STAT5B* variant was not restricted to one TCR clone, as the top TCR γδ clone frequency in P1 was 6.7% (Table 1 and Table 3). The identified STAT5B missense variant is located within the STAT5B SH2 domain (Fig. 1, G and H). This variant has been previously shown to cause STAT5 GOF, displaying enhanced DNA binding, while having a less significant impact on STAT5 phosphorylation (26). We confirmed that the c.A1994T mutation was expressed at the mRNA level through Sanger sequencing of a PCR product generated from cDNA of sorted TCR γδ cells (Fig. 1 F). The STAT5B mutation was not detected by Sanger sequencing in various enriched myeloid lineage populations (monocytes, granulocytes, and eosinophils) of P1 (Fig. S1, E–G).

**Table 4.** IEI gene variants detected by WES of TCR αβ and TCR γδ cells

Chr	rsID	Zygosity	Gene	cDNA	Amino acid	Allele frequency	CADD	Cells (TCR)
2	rs147471840	het	*TTC7A*	c.G563A	p.R188H	0.0007813	29.1	αβ, γδ
4	rs151048521	het	*SH3BP2*	c.C1825T	p.R609W	0.00005418	35	αβ, γδ
8	rs113671330	het	*VPS13B*	c.A9424G	p.S3142G	0.009001	18.7	αβ, γδ
17	–	het	*STAT5B*	c.A1994T	p.Y665F	–	24.5	γδ
19	rs147442318	het	*TYK2*	C2985T	p.I995I	0.0002916	16.55	αβ, γδ
19	rs757581005	het	*TYK2*	c.C370T	p.R124C	0.00001193	33	αβ, γδ
19	rs145148929	het	*TICAM1*	c.C479T	p.S160F	0.002158	24	αβ, γδ
22	rs11548656	het	*TNFRSF13C*	c.C246T	p.P82P	0.02566	15.19	αβ, γδ

CADD score, combined annotation-dependent depletion score; Chr, Chromosomers; rsID, reference single nucleotide polymorphism cluster identification.

WES analysis of gDNA from flow cytometry–sorted TCR γδ cells of one other patient (cohort number 86) detected neither STAT3 nor STAT5 mutations that could have explained the expansion of TCR γδ cells. In the remaining three other patients, we have as of yet only performed targeted Sanger sequencing of the corresponding STAT5B region using gDNA from bulk PBMCs, without the detection of a somatic mutation (Table 1). Bulk sequencing may, however, miss low-frequency TCR γδ cell–restricted somatic mutations.

To validate STAT5B activity in primary T cells of P1, we measured STAT5 phosphorylation in TCR αβ vs. TCR γδ cells isolated from PBMCs from P1 vs. five healthy controls (HCs) using flow cytometry (Fig. 1, I and J). Sorted TCR αβ and TCR γδ cells were rested overnight, and stimulated (or not) with recombinant IL-2 for 30 min. After IL-2 stimulation, P1-derived TCR γδ cells showed a significantly higher percentage of phospho-STAT5^high^ (pSTAT5) cells compared with HC, while TCR αβ cells showed no significant difference in STAT5 phosphorylation (Fig. 1 J). Similar results were observed for the measurements of pSTAT5 Median fluorescence intensity (MFI) (Fig. S1 H). In the absence of IL-2 stimulation, TCR αβ cells and TCR γδ cells showed no differences in pSTAT5 MFI (Fig. S1 I). These data indicate that the missense mutation drives STAT5 hyperphosphorylation in TCR γδ cells upon IL-2 stimulation.

### Phenotypic characterization of peripheral TCR Vδ2 cells from P1

**Figure S2.**
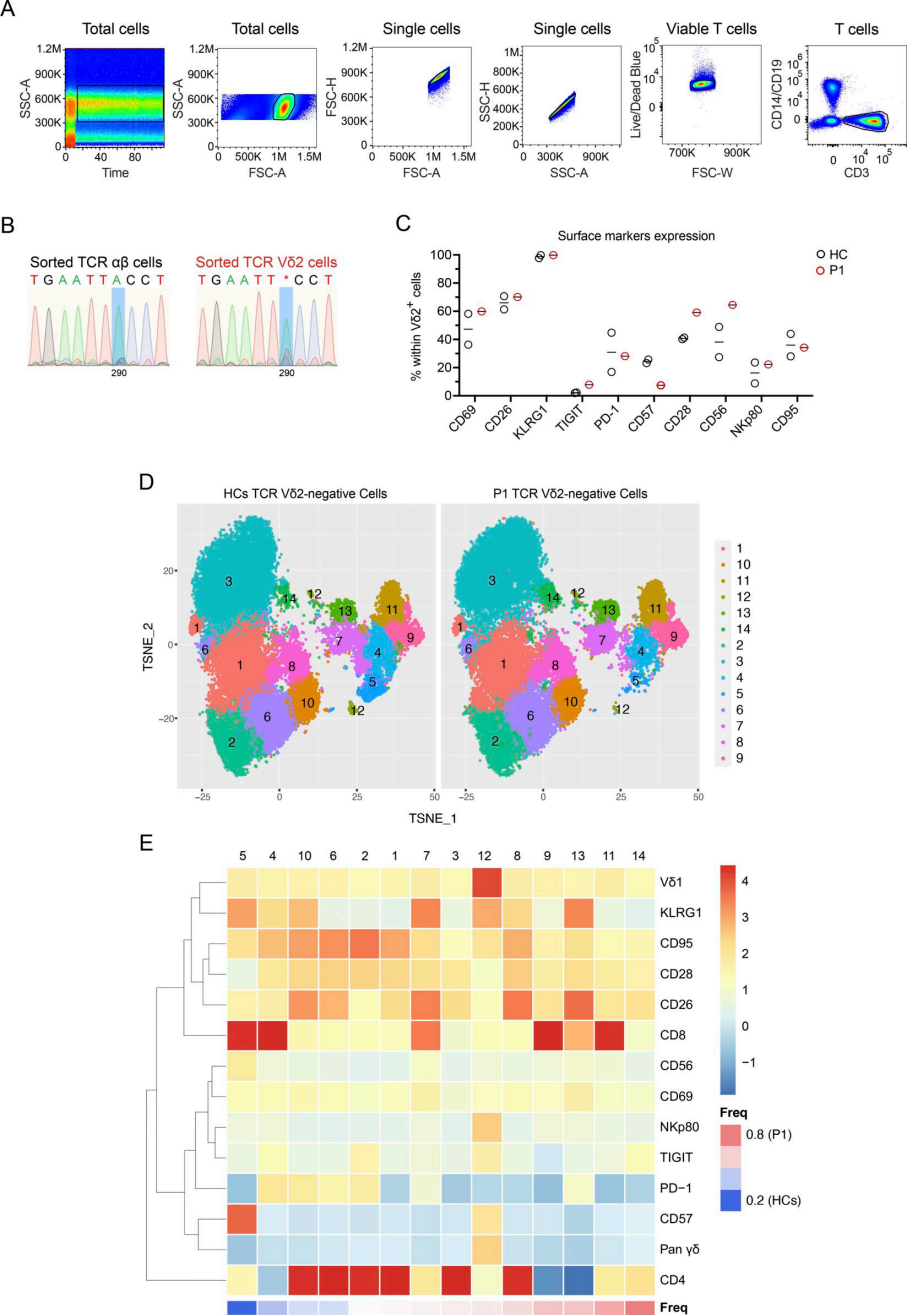
**Characterization of TCR Vδ2–negative cells from P1. (A)** Gating strategy used to characterize T cells from P1 and HCs by multicolor flow cytometry analysis. The initial events of the acquisition were excluded due to stream instability observed during the first seconds of acquisition (SSC-A/Time). Lymphocytes were sequentially gated according to FSC-A/SSC-A, followed by singlets (FSC-A/FSC-H and SSC-A/SSC-H), and viable cells (FSC-W/excluded with LIVE/DEAD Blue). Then, CD3 cells were selected (CD3^+^/CD14^−^CD19^−^) and analyzed. **(B)** Sanger sequencing of p.Y665F STAT5B region amplified from cDNA of PBMC-derived freshly sorted TCR αβ (left) and TCR Vδ2 (right) cells. **(C)** Summary of CD69, CD26, KLRG1, TIGIT, PD-1, CD57, CD28, CD56, NKp80, and CD95 expression on PBMC-derived TCR Vδ2 cells from two HCs (black circles) and P1 (red circles). The data correspond to one experiment. **(D)** t-distributed stochastic neighbor embedding (t-SNE) of TCR Vδ2–negative cells from two HCs and P1 distributed in clusters 1 to 14 according to TCR Vδ1, KLRG1, CD95, CD28, CD26, CD8, CD56, CD69, NKp80, TIGIT, PD-1, pan-γδ, and CD4 expression. **(E)** Heatmap of clustering characterization of TCR Vδ2–negative cells from two HCs and P1 according to the expression of TCR Vδ1, KLRG1, CD95, CD28, CD26, CD8, CD56, CD69, NKp80, TIGIT, PD-1, pan-γδ, and CD4.

**Figure 2.**
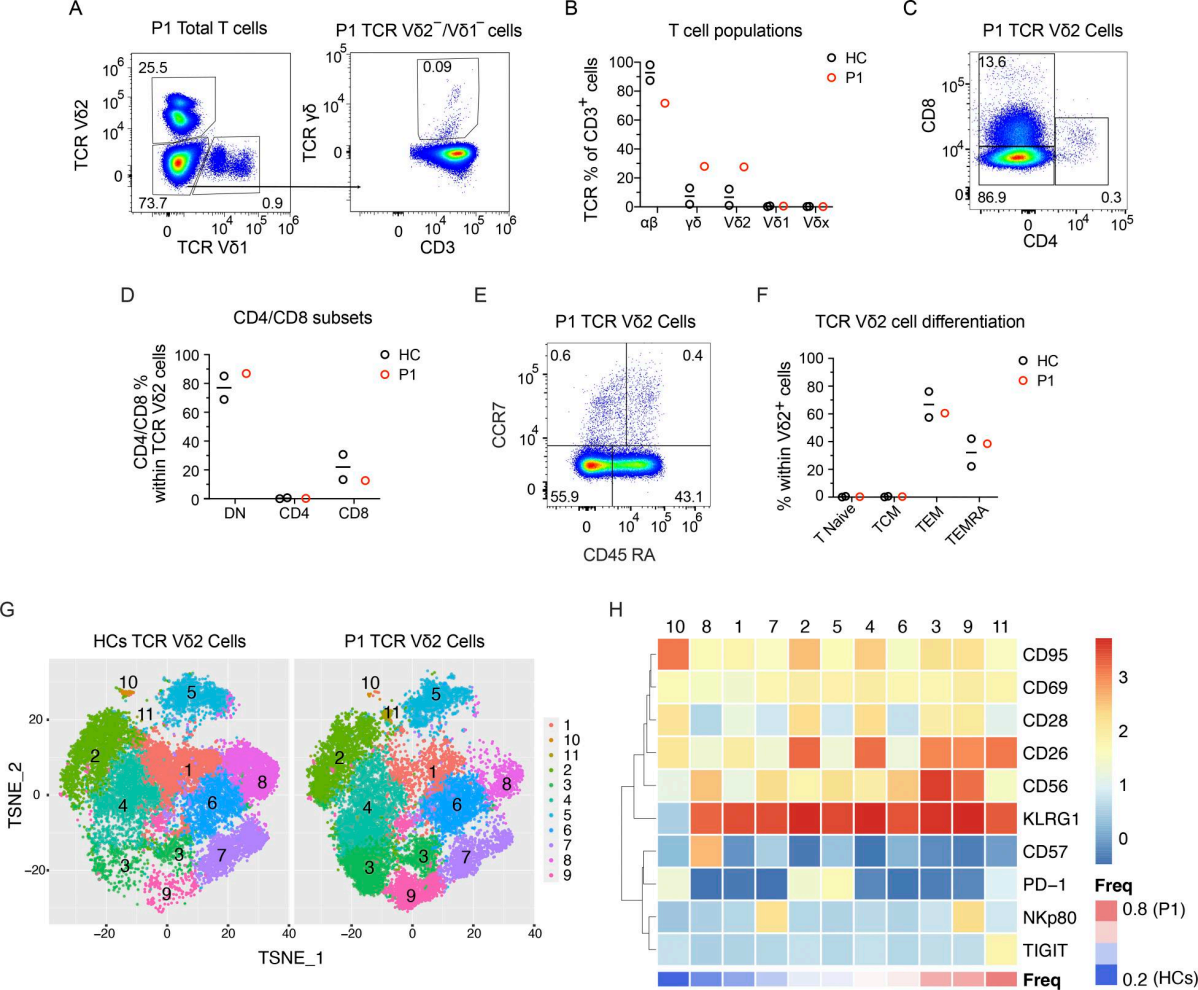
**TCR γδ cells of P1 predominantly express the Vδ2 chain. (A)** Representative flow cytometry plot of TCR Vδ2 vs. TCR Vδ1 (left plot) on total T cells derived from PBMCs of P1 and CD3 vs. TCR γδ (right plot) on TCR Vδ2^–^/Vδ1^–^ cells. **(B)** Summary of TCRs expressed on PBMC-derived T cells from two HCs (black circles) and P1 (red circles). **(C)** Representative flow cytometry plots of CD4 and CD8 expression on TCR Vδ2 cells derived from P1 PBMCs. **(D)** Summary of CD4 and CD8 expression on PBMC-derived TCR Vδ2 cells from two HCs (black circles) and P1 (red circles). DN: CD4^−^CD8^–^ double negative. **(E)** Representative flow cytometry plots of CCR7 and CD45RA expression on TCR Vδ2 cells derived from P1 PBMCs. **(F)** Summary of CCR7 and CD45RA expression on PBMC-derived TCR Vδ2 cells from two HCs (black circles) and P1 (red circles). T naive: naive T cell; TCM: central memory T cell; TEM: effector memory T cell; TEMRA: effector memory T cell CD45RA-positive. **(G)** t-SNE of TCR Vδ2 cells from two HCs and P1 distributed in clusters 1–11 according to CD95, CD69, CD28, CD26, CD56, KLRG1, CD57, PD-1, NKp80, and TIGIT expression. **(H)** Heatmap of clustering characterization of TCR Vδ2 cells from two HCs and P1 according to the expression of the CD95, CD69, CD28, CD26, CD56, KLRG1, CD57, PD-1, NKp80, and TIGIT.

T cells isolated from PBMCs of P1 and two HCs were further characterized using multicolor flow cytometry (gating strategy displayed in Fig. S2 A). This confirmed the reduced frequency of TCR αβ cells and an increased frequency of TCR γδ cells in P1, and demonstrated that the majority of TCR γδ cells were TCR Vδ2 cells (Fig. 2, A and B). We isolated TCR Vδ2 from P1-derived PBMCs and confirmed the presence of the mutated STAT5B in the TCR Vδ2 subset (Fig. S2 B). TCR Vδ2 cells of both P1 and HCs primarily displayed a CD4^−^CD8^−^ phenotype, representing ∼80%, while the remaining (∼20%) expressed only CD8 (Fig. 2, C and D). The differentiation of the TCR Vδ2 subset was assessed based on the expression of CD45RA and CCR7 (Fig. 2 E). TCR Vδ2 cells from both P1 and HCs were primarily T effector memory (TEM) or T effector memory re-expressing CD45RA (TEMRA), with frequencies of ∼65 and 30%, respectively (Fig. 2 F). 10 different surface markers were analyzed for the TCR Vδ2 population and were used in clustering analysis (Fig. S2 C; and Fig. 2, G and H).

The TCR Vδ2 cells from both P1 and HCs exhibited elevated KLRG1 levels, indicating that they were antigen-experienced (Fig. S2 C). The activation markers CD69 and CD26, the inhibitory molecule PD-1, the apoptosis-inducing mediator CD95, and the NK marker NKp80 were similarly expressed by TCR Vδ2 cells from both groups (Fig. S2 C). Instead, the NK marker CD56 and the costimulatory molecule CD28 were higher in TCR Vδ2 cells from P1 compared with HCs. Additionally, TIGIT was expressed in a subset of TCR Vδ2 cells from P1 (Fig. S2 C). Finally, CD57 was more abundantly expressed in TCR Vδ2 cells from HCs compared with P1, where it was nearly absent (Fig. S2 C). The expression of CD57 on lymphocytes may indicate reduced proliferative capacity (27), linking the CD57^low^ expression in P1-derived TCR Vδ2 cells to the high frequencies observed in the blood of P1 and to the STAT5B GOF mutation.

The clustering analysis of these markers revealed that clusters 10 (KLRG1^−^, CD28^+^, CD95^+^, PD-1^+^), 8 (KLRG1^+^, CD56^+^, CD57^+^), and 1 (KLRG1^+^) were more prevalent in HCs than in P1. Cluster 10 defines a population of PD-1^+^ TCR Vδ2 cells with high cytotoxic capacity linked to CD95 expression. However, the overall presence of this cluster was very low in both HCs and P1. Conversely, cluster 8, representing CD56^+^ TCR Vδ2 cells with low proliferation capacity related to CD57 expression, and cluster 1, which showed no expression of inhibitory or activation markers, constituted a substantial part of the TCR Vδ2 cells found in the periphery (Fig. 2, G and H). In summary, these phenotypic data suggest a decrease in nonactivated TCR Vδ2 cells, characterized by lower proliferative capacity, in P1. The enriched clusters identified in P1, including clusters 3 (KLRG1^+^, CD26^bright^, CD56^bright^), 9 (KLRG1^+^, CD26^bright^, CD56^bright^, NKp80^+^), and 11 (KLRG1^+^, CD26^bright^, TIGIT^+^), indicate an enrichment of activated TCR Vδ2 cells with an NK-like phenotype (Fig. 2, G and H).

As a control, the clustering of the TCR Vδ2^−^ cells showed no significant differences in the measured markers. Cluster 14, which consists of nonactivated TCR γδ–negative cells, and cluster 5, featuring CD8^+^ and CD57^+^ TCR γδ–negative cells, were enriched in P1 and HCs, respectively. However, these clusters constitute only a small fraction of the TCR Vδ2–negative cells (Fig. S2, D and E).

### P1-derived TCR Vγ9Vδ2 cells demonstrate enhanced proliferation and altered cytokine profile in response to pAgs

**Figure 3.**
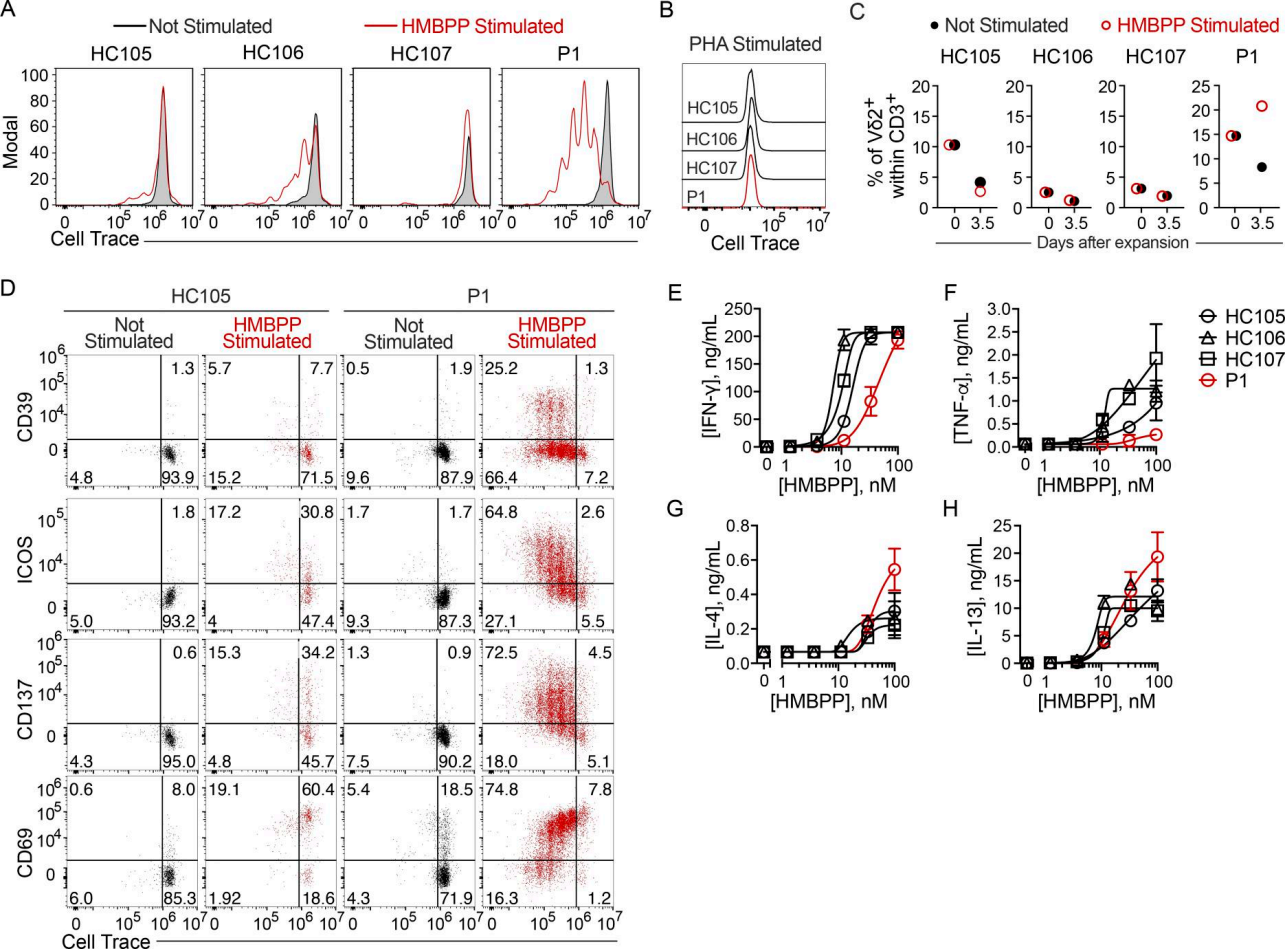
**P1-derived TCR Vδ2 cells expand more efficiently in the presence of HMBPP. (A)** Proliferation of PBMC-derived TCR Vδ2 cells after 3.5 days in the presence (red) or absence (black) of a suboptimal dose of HMBPP (0.4 nM). Representative cell trace histograms represent samples from HC105, HC106, HC107, and P1. **(B)** Proliferation of PBMC-derived TCR Vδ2 cells after 3.5 days in the presence of 1 µg/ml of phytohemagglutinin. Representative cell trace histograms are shown for samples from HC105, HC106, HC107 (black), and P1 (red). **(C)** Percentages of TCR Vδ2 cells within T cells at days 0 and 3.5 in the presence or absence of HMBPP (0.4 nM). The data correspond to one experiment. **(D)** Representative flow cytometry plots of CD39, ICOS, CD137, and CD69 expression on TCR Vδ2 cells from P1 and HC105 expanded in the presence (red) or absence (black) of HMBPP (0.4 nM). **(E–H)** Activation of TCR Vδ2 cell lines derived from HC105, HC106, HC107 (black), and P1 (red) challenged with THP-1 cells exposed to increasing doses of HMBPP. Dose–response curves of (E) IFN-γ, (F) TNF-α, (G) IL-4, and (H) IL-13 release mean ± SD of triplicate independent cultures. Representative plot of one of two experiments.

**Figure S3.**
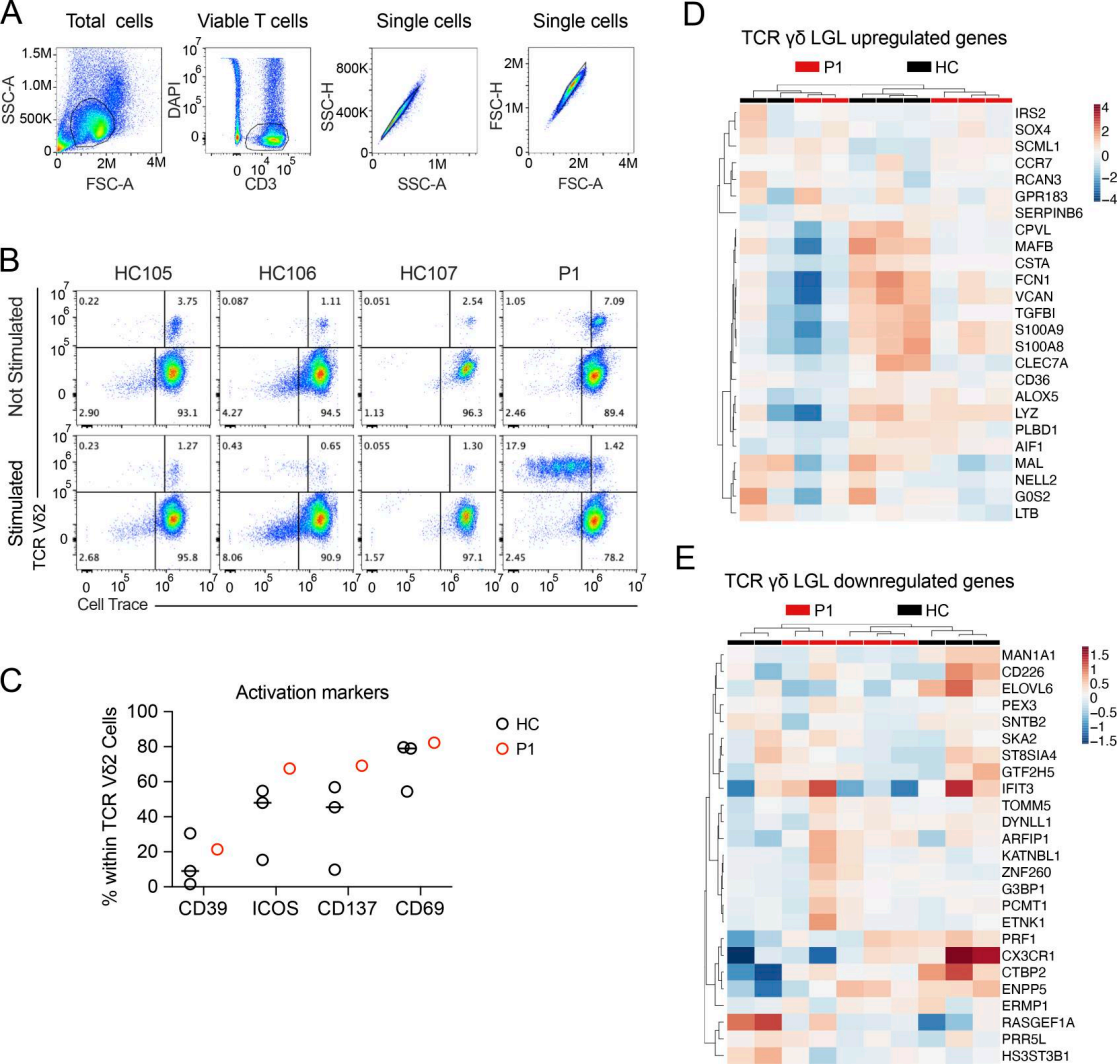
**Proliferation and activation assays of TCR Vδ2 cells challenged with HMBPP in P1 vs. HCs. (A)** Gating strategy to assess proliferation and activation of T cells, which were freshly isolated from PBMCs and challenged with HMBPP (0.4 nM). Lymphocytes were sequentially gated on FSC-A/SSC-A, followed by viable CD3 cells (CD3^+^/LIVE/DEAD excluded with DAPI). Singlets were selected on FSC-A/FSC-H and SSC-A/SSC-H, and the expansion of TCR Vδ2–positive cells was determined using a cell trace. **(B)** Flow cytometry plots of TCR Vδ2 cells from HC105, HC106, HC107, and P1 expanded for 3.5 days in the presence or absence of HMBPP (0.4 nM). PBMCs were cultured with or without HMBPP, and the expansion of total T cells was analyzed. **(C)** Summary of TCR Vδ2 cells from HCs (black circles) and P1 (red circles) that upregulated CD39, ICOS, CD137, and CD69 after 3.5 days of being challenged with HMBPP (0.4 nM). Medians are represented in each condition. The data correspond to one experiment. **(D and E)** Heatmap of TCR γδ T-LGL leukemia–related genes reported to be (D) upregulated or (E) downregulated. TCR γδ cells from P1 were isolated at five different time points.

STAT5 phosphorylation is essential for T cell proliferation (28). To investigate whether the STAT5B GOF mutation found in TCR Vγ9Vδ2 cells from P1 enhances their ability to proliferate in vitro, we stimulated PBMCs from three HCs and P1 with a suboptimal dose of the pAg (E)-4-hydroxy-3-methyl-but-2-enyl pyrophosphate (HMBPP) (0.4 nM). This low dose caused a distinct proliferation of TCR Vδ2 cells from P1, but not from HCs (Fig. 3 A). No other T cells were expanded from the HMBPP stimulation (Fig. S3, A and B). Despite differences in proliferative capacity under suboptimal HMBPP stimulation, TCR Vδ2 cells from both P1 and HCs exhibited similar proliferative potential when stimulated with PHA (Fig. 3 B), indicating that the response to TCR stimulation was enhanced in P1, while the reaction to other PHA-stimulated receptors was not. In all three HCs, suboptimal HMBPP doses did not alter the in vitro frequencies of TCR Vδ2 cells. However, in P1, it increased the percentage of TCR Vδ2 cells within CD3^+^ cells from 15 to 21% (Fig. 3 C). When activation marker expression was assessed, CD69, CD137, and ICOS were detected on most proliferating cells after just one cell division (Fig. 3 D and Fig. S3 C). CD39 was expressed in only about one third of proliferating cells and appeared after at least two divisions (Fig. 3 D and Fig. S3 C).

The activation of TCR Vγ9Vδ2 cell lines derived from P1 vs. HCs was further evaluated by measuring cytokine release. HMBPP stimulation caused a greater dose-dependent secretion of IFN-γ (Fig. 3 E) and TNF-α (Fig. 3 F) from HC lines compared with P1, indicating a preferential Th1 response in TCR Vγ9Vδ2 cell lines derived from HCs. In contrast, the P1-derived TCR Vγ9Vδ2 cell line shows a trend toward secreting more IL-4 (Fig. 3 G) and IL-13 (Fig. 3 H) than HC lines in response to 100 nM HMBPP. This suggests a distinct Th2-skewed cytokine profile in P1-derived TCR Vγ9Vδ2 cells, consistent with the clinical and laboratory phenotype.

### Transcriptome of P1’s STAT5 GOF–mutated TCR γδ cells

**Figure 4.**
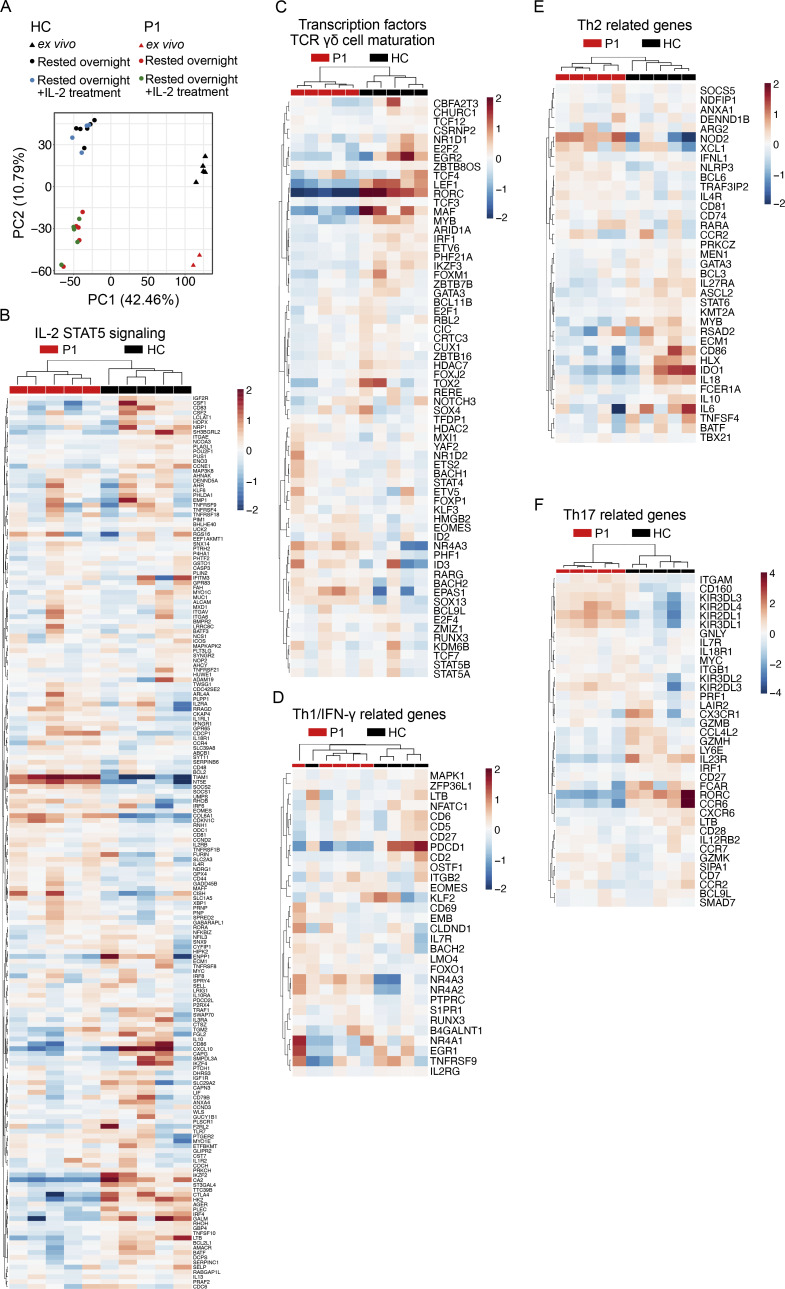
**Differential gene expression in TCR γδ cells from P1 vs. HCs. (A)** PCA of RNA-seq data comparing P1 with HC TCR γδ cells. The four clusters identified correspond to TCR γδ cells from P1 (red) and HCs (blue) in overnight-rested (dots) or ex vivo (triangles) samples. Cells treated with IL-2 for 30 min from HC (blue dots) and P1 (green dots) clusters were compared with IL-2–untreated cells. Samples for RNA-seq were collected from five HCs and from P1 at five time points; RNA-seq was performed in a single experiment. **(B–F)** Heatmaps of differentially expressed genes related to (B) the IL-2-STAT5 signaling pathway, (C) transcription factors and TCR γδ cell maturation, (D) Th1/IFN-γ, (E) Th2, and (F) Th17 profile. TCR γδ cells from P1 were isolated at five different time points. PCA, principal component analysis.

To define the gene expression profile and differentiation program of the STAT5 GOF TCR γδ cells, we sorted TCR γδ cells from P1 and HCs and performed RNA sequencing (RNA-seq). The PCA showed that TCR γδ cells from P1 and HCs were transcriptionally distinct both in ex vivo and in in vitro overnight-rested TCR γδ cells (Fig. 4 A).

We also performed RNA-seq on TCR γδ cells from P1 and HCs, treated or not with IL-2 for 4 h before RNA extraction. PCA revealed that untreated and IL-2–treated TCR γδ cells clustered together for both P1 and HCs, indicating that IL-2 stimulation did not result in significant overall differential gene expression (Fig. 4 A).

Since STAT5 is essential for the IL-2 signaling pathway (29), we initially examined related genes (Fig. 4 B). Classical STAT5B target genes, such as IL-2RB, SOCS2, and CISH, were upregulated in P1 (Fig. 4 B). Compared with HCs, P1-derived TCR γδ cells showed significantly increased expression of genes involved in T cell survival (*NT5E*, *COL6A1*) (30, 31), expansion (*CDKN1C*) (32), migration (*TIAM1*) (33), suppression of T cell response (*COL6A1*) (34), and inhibition of the JAK-STAT pathway (*CDCP1*) (35). In contrast, TCR γδ cells from P1 had reduced expression of genes that coordinate inhibition of cell proliferation and migration (*SH3BGRL2*) (36), T cell activation (*ENPP1*, *IKZF2*, *CTL4*, *HK2*, *IRF4*, *TNFSF10*) (37, 38, 39, 40, 41, 42), and exhaustion (*ENPP1*, *IRF4*) (37, 43). Notably, a published transcriptomic signature of TCR γδ T-LGL leukemia (44) was assessed in P1 vs. HCs samples. The top dysregulated genes from this published γδ T-LGL leukemia signature did not cluster P1 vs. HC samples (Fig. S3, D and E).

On the one hand, these transcriptomic data indicate that STAT5-mutated TCR γδ cells are more prone to proliferate. On the other hand, analysis of transcription factor genes (45) in P1’s TCR γδ cells showed decreased expression of genes involved in T cell activation (*EGFR2*) (46), exhaustion (MAF) (47), memory cell generation (*LEF1*) (48), and the induction of the Th17 program (*RORC*) (49) (Fig. 4 C). Additionally, upregulated NR4A3 expression might reduce cytokine production during the effector stage (50) (Fig. 4 C). Next, we focused the analysis on the functional differentiation programs of TCR γδ cells by comparing the transcription of genes associated with the Th1/IFN-γ, Th2, and Th17 effector functions (51) (Fig. 4, D and F). The analysis revealed minimal differences in the Th1/IFN-γ and Th2 gene sets (Fig. 4, D and E). In P1, there were a decrease in the expression of the Th1 cell activation marker gene *PDCD1* (Fig. 4 D) and an increase in the expression of the Th2-related gene *NOD2*, which plays a role in regulating T cell activation (52) (Fig. 4 E).

Interestingly, major differences were observed in genes associated with Th17 differentiation. TCR γδ cells derived from P1 showed higher transcripts for inhibitory NK markers (*KIR3DL3*, *KIR3DL1*, *KIR2DL4*, *KIR2DL1*) (53), and reduced expression of Th17-associated genes (*IL23R*, *RORC*, *CCR6*, *MAF*) (54) (Fig. 4 F).

### TCR γδ cells from P1 display a repressed Th17 and augmented Th9 signature

**Figure 5.**
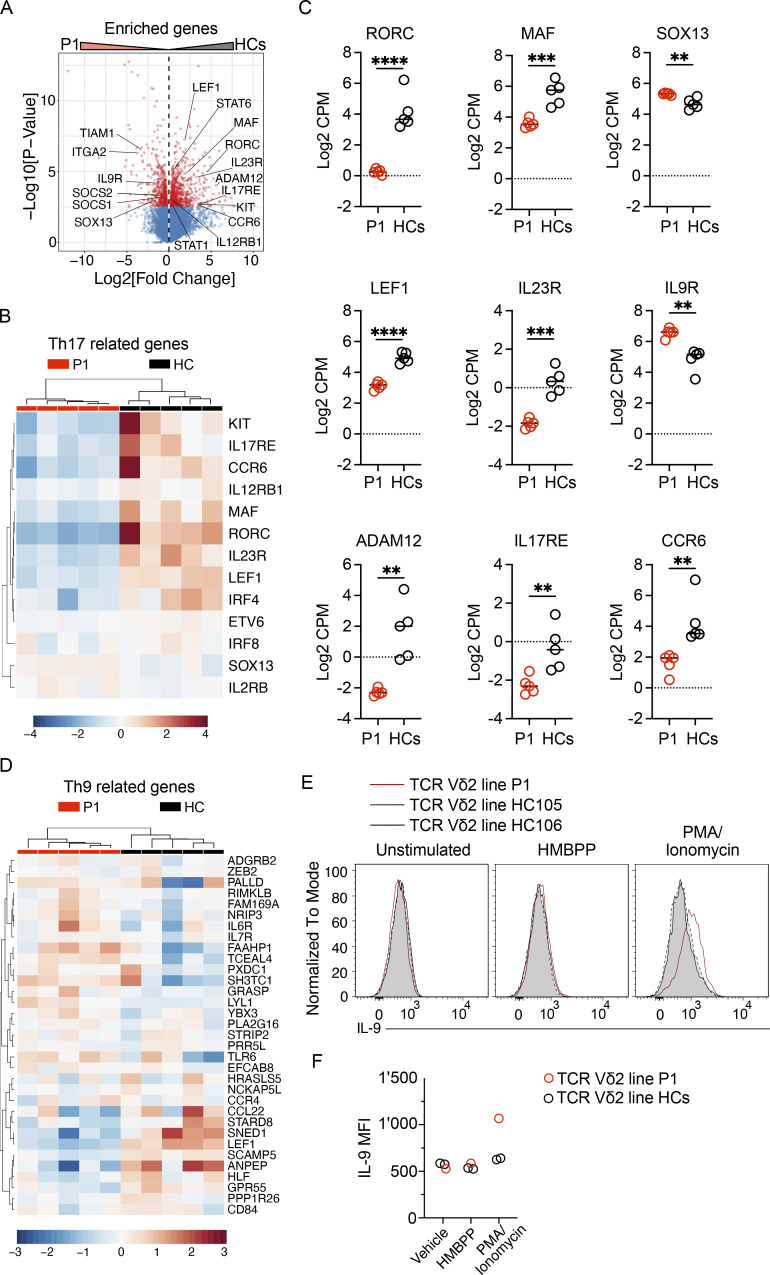
**P1-derived TCR γδ cells express fewer Th17-related genes than HCs and have a Th1 profile. (A)** Volcano plot of genes differentially expressed between TCR γδ cells derived from P1 or HCs. Genes represented as red dots have a P <0.05, and genes represented as blue dots have a P >0.05. Marked genes are linked to TH17 differentiation (*RORC*, *MAF*, *SOX13*, *IL23R*, *IL12RB1*, *IL17RE*, *KIT*, CCR6, LEF1, TIAM1, ITGA2) or to STAT pathways (STAT1, STAT6, SOCS1, SOCS2, IL-9R). **(B)** Heatmap of genes involved in Th17 differentiation and STAT5B regulation (RORC, MAF, CCR6) differentially expressed between P1 and HCs. **(C)** mRNA expression level of RORC, MAF, SOX13, LEF1, IL-23R, IL-9R, ADAM12, IL-17RE, and CCR6 from overnight-rested P1 (red circles)– and HC (black circles)–derived TCR γδ cells. Expression levels are shown as Log2 CPM. Normality was assessed using the Shapiro–Wilk test. For normally distributed data, an unpaired *t* test was used. For data without a normal distribution, the Mann–Whitney test was used. **P < 0.01, ***P < 0.001, ****P < 0.0001. **(D)** Heatmap of genes related to the Th9 profile differentially expressed between P1 and HCs. **(E)** Activation of TCR Vδ2 cell lines challenged for 12 h with THP-1 cells exposed to a vehicle (left plots) or 4 nM of HMBPP (middle plots). T cells were also exposed to PMA/ionomycin to induce strong cell activation (right plots). Histograms of intracellular staining of IL-9 produced by TCR Vδ2 cells from HC105 (dashed black line), HC106 (black line), and P1 (red line). The data correspond to one experiment. **(F)** Intracellular cytokine production of IL-9 by TCR Vδ2 cells from P1 (red circles) and HCs (black circles). MFI values are represented for IL-9. CPM, count per million reads.

Next, we characterized genes known to be involved in Th17 cell function to further evaluate the differentiation signature of the TCR γδ cells from P1 (51) (Fig. 5 A).

**Figure S4.**
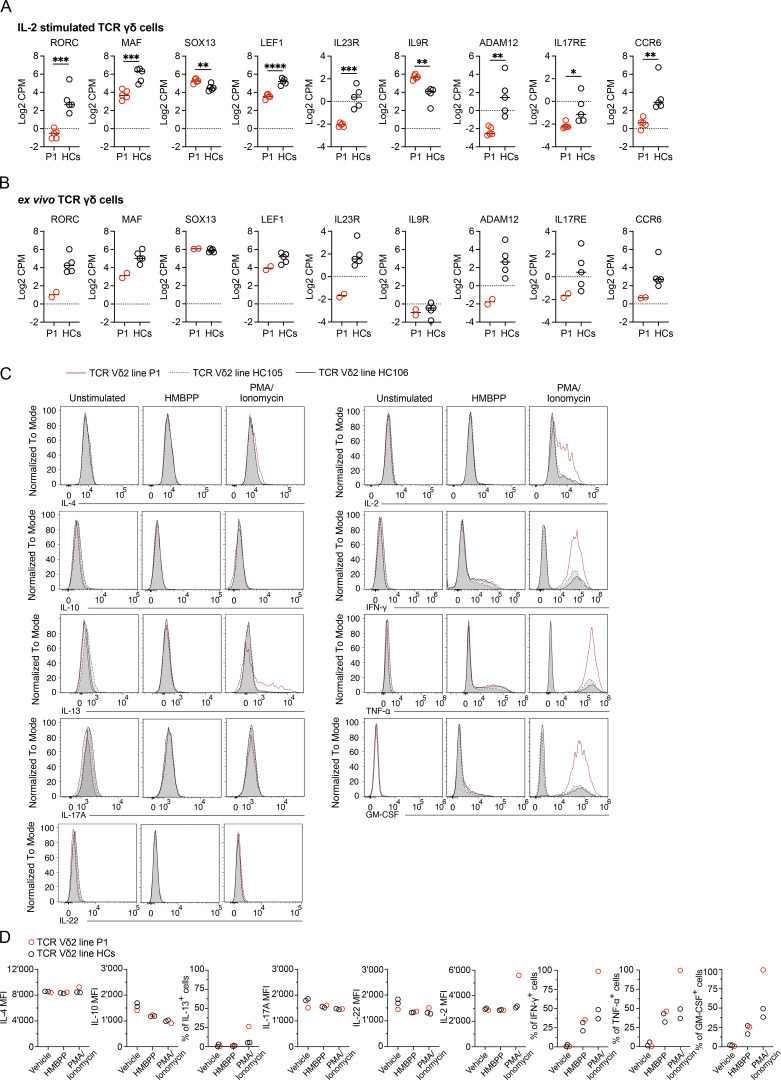
**Comparison of gene expression in TCR γδ cells and cytokine profiling in P1 vs. HCs. (A)** mRNA expression level of RORC, MAF, SOX13, LEF1, IL-23R, IL-9R, ADAM12, IL-17RE, and CCR6 from P1 (red circles)– and HC (black circles)–derived TCR γδ cells stimulated with IL-2 (100 U/ml) for 4 h. Expression levels are shown as Log2 CPM. Normality was assessed using the Shapiro–Wilk test. For normally distributed data, an unpaired *t* test was used. For data that were not normally distributed, the Mann–Whitney test was used. *P < 0.05, **P < 0.01, ***P < 0.001, ****P < 0.0001. **(B)** mRNA expression level of RORC, MAF, SOX13, LEF1, IL-23R, IL-9R, ADAM12, IL-17RE, and CCR6 from ex vivo P1 (red circles)– and HC (black circles)–derived TCR γδ cells. Expression levels are shown as Log2 CPM. **(C)** Activation of TCR Vδ2 cell lines challenged for 12 h with THP-1 cells exposed to a vehicle (left plots) or 4 nM of HMBPP (middle plots). T cells were also exposed to PMA/ionomycin to induce strong cell activation (right plots). Histograms of intracellular staining of IL-4, IL-10, IL-13, IL-17A, IL-22, IL-2, IFN-γ, TNF-α, and GM-CSF cytokines produced by TCR Vδ2 cells from HC105 (dashed black line), HC106 (black line), and P1 (red line). **(D)** Summary of intracellular cytokine production of IL-4, IL-10, IL-17A, IL-22, and GM-CSF by TCR Vδ2 cells from P1 (red circles) and HCs (black circles). MFI values are represented for IL-4, IL-10, IL-17A, IL-22, and IL-2. Percentages are represented for IL-13^+^, IFN-γ^+^, TNF-α^+^, and GM-CSF^+^ cells. The data correspond to one experiment. CPM, count per million reads.

This analysis revealed a decline in several key transcription factors characteristic of Th17 cells (*RORC*, *MAF*, *LEF1*) and other genes associated with Th17 cells (*IL17RE*, *KIT*, *IL23R*, *CCR6*, *IL12RB1*, *ADAM12*) (Fig. 5, A and B). These genes were significantly downregulated in TCR γδ cells from P1 compared with HCs in both unstimulated (Fig. 5 C) and IL-2–stimulated (Fig. S4 A) TCR γδ cells. The repression pattern of these genes in P1 was similar in ex vivo TCR γδ cells (Fig. S4 B). Moreover, the negative regulators of STAT5 hyperactivity, SOCS1 and SOCS2, were elevated in P1, suggesting a compensatory response in STAT5 GOF mutant TCR γδ cells (Fig. 5 A).

The analysis of genes differentially expressed in TCR γδ cells derived from P1 vs. HCs showed an increased expression of IL-9R in P1 (Fig. 5, A and C).

Next, we examined the profile of intracellular cytokines produced by TCR Vδ2 cells from P1 compared with HCs. We found no differences in the production of Th1 cytokines (IL-2, IFN-γ, TNF-α, GM-CSF), Th2 cytokines (IL-4, IL-10, IL-13), Th9 cytokine (IL-9), and Th17 cytokines (IL-17A, IL-22) upon HMBPP stimulation. However, significant differences emerged between the lines after PMA/ionomycin exposure. P1-derived TCR Vδ2 cells showed higher intracellular levels of all measured Th1 cytokines (IL-2, IFN-γ, TNF-α, GM-CSF) compared with HCs. Additionally, Th2 cytokines IL-4 and IL-13 were slightly increased in P1-derived TCR Vδ2 cells (Fig. S4, C and D). Even though the overall Th9 transcriptional profile (55) was not strongly upregulated in P1 compared with HCs (Fig. 5 D), we detected increased IL-9 (Th9) production upon PMA/ionomycin exposure. The cytokines IL-10, IL-17A, and IL-22 were not detected in any of the tested stimulation conditions (Fig. 5, E and F; and Fig. S4, C and D).

### JAK inhibition reduces hyperinflammation and STAT5 phosphorylation in P1, associated with improved clinical manifestations and decreasing γδ T cells

**Figure 6.**
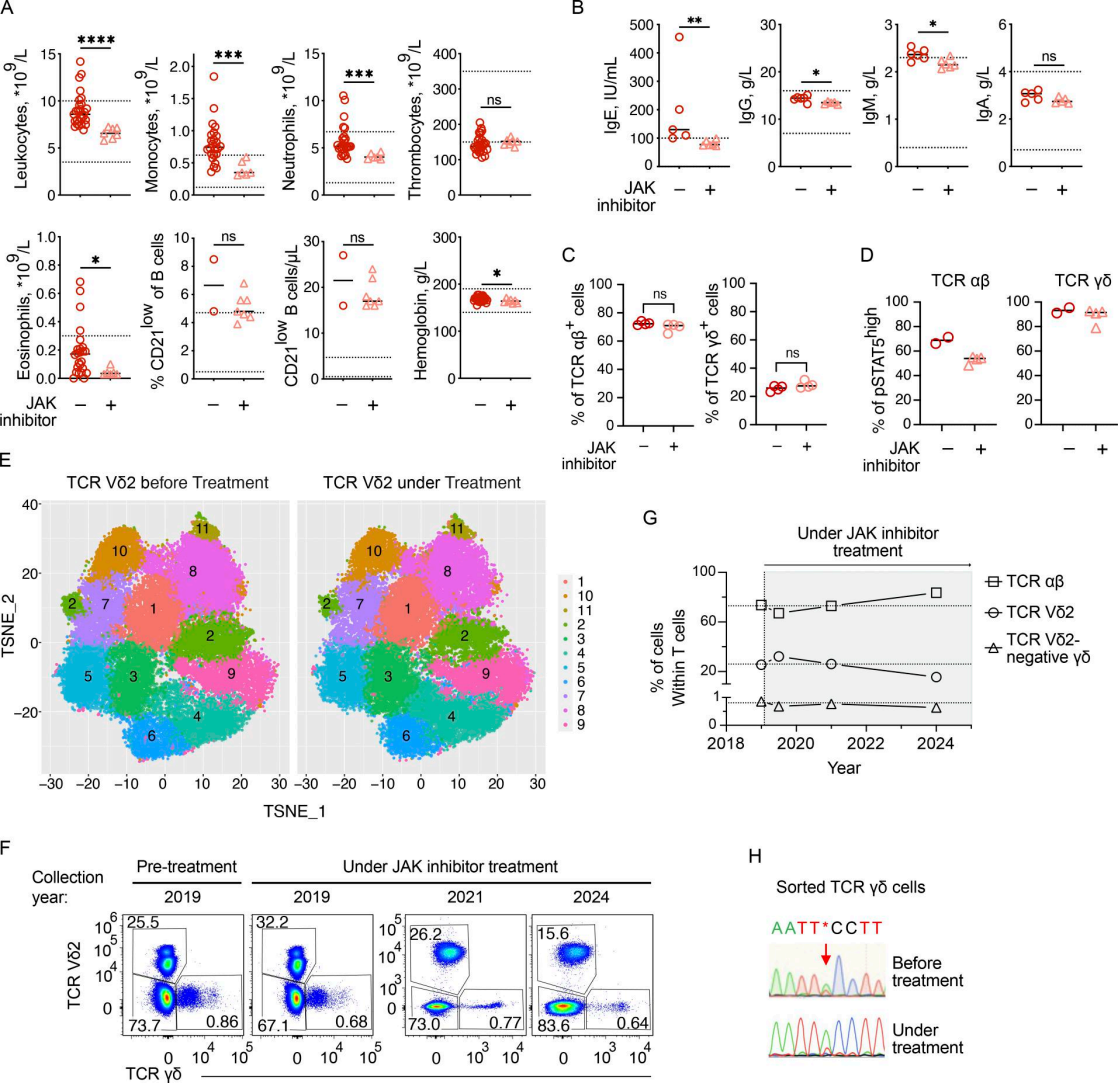
**Clinical and immunophenotypic characterization of P1 before and under JAK inhibition. (A)** Absolute numbers of leukocytes, monocytes, neutrophils, thrombocytes, eosinophils, CD21^low^ B cells, hemoglobin, and percentage of CD21^low^ B cells in the blood before and under JAK inhibition treatment. Normality was assessed using the Shapiro–Wilk test. For normally distributed data, an unpaired *t* test was used. For data that were not normally distributed, the Mann–Whitney test was used. *P < 0.05, ***P < 0.0001, ****P < 0.0001. **(B)** IgE, IgG, IgM, and IgA levels in the blood before and under JAK inhibition treatment. Mann–Whitney test. Normality was assessed using the Shapiro–Wilk test. For normally distributed data, an unpaired *t* test was used. For data that were not normally distributed, the Mann–Whitney test was used. ns: not significant, *P < 0.05, **P < 0.01. **(C)** Summary of T cells from P1 expressing TCR αβ (left) or TCR γδ (right) before and under treatment with JAK inhibitor (within 6 mo after start of treatment). Scatter plots of percentages and the median of four independent measurements. Mann–Whitney test. ns: not significant. **(D)** Summary of percentages of pSTAT5 in TCR αβ (left) or TCR γδ (right) cells isolated from P1 before and under treatment with JAK inhibitor after stimulation with IL-2 (100U/ml) for 4 h. **(E)** TSNE of TCR Vδ2 cells from P1 before and under treatment with JAK inhibitor distributed in clusters 1–11 according to CD95, CD28, CD26, CD56, KLRG1, NKp80, TIGIT, PD-1, and CD57 expression. **(F)** Representative flow cytometry plot of TCR Vδ2 vs. TCR γδ on total T cells derived from PBMCs of P1 at different collection dates since the beginning of JAK inhibition treatment. **(G)** Percentages of TCR γδ, TCR Vδ2, and TCR αβ in PBMCs of P1 at different collection dates since the beginning of JAK inhibition treatment. The data correspond to one experiment. **(H)** Sanger sequencing of p.Y665F STAT5B region amplified from cDNA of freshly sorted TCR γδ from P1 before treatment (top panel) or under treatment (year of analysis = 2021; bottom panel) with JAK inhibitor.

JAK inhibitors have been reported to successfully treat autoimmune manifestations associated with germline GOF mutations in STAT3 or somatic GOF mutations in STAT5 (56, 57, 58). To our knowledge, JAK inhibition in patients with TCR γδ cell–specific STAT5 GOF has not yet been documented. We started therapy by administering 4 mg/day of the JAK inhibitor baricitinib under careful clinical, laboratory, and molecular monitoring. Treatment of P1 with the JAK inhibitor markedly reduced the severity and frequency of oral aphthous lesions. It normalized the patient’s reported susceptibility to airway infections and the numbers of monocytes, eosinophils, and neutrophils in the blood (Fig. 6 A). Furthermore, we observed reductions in serum IgE and IgM levels (Fig. 6 B). Initially, the percentages of CD21^low^ B cells decreased, but did not return to normal reference levels (Fig. 6 A). Hemoglobin levels (Fig. 6 A) and serum IgG and IgA concentrations (Fig. 6 B) remained normal.

**Figure S5.**
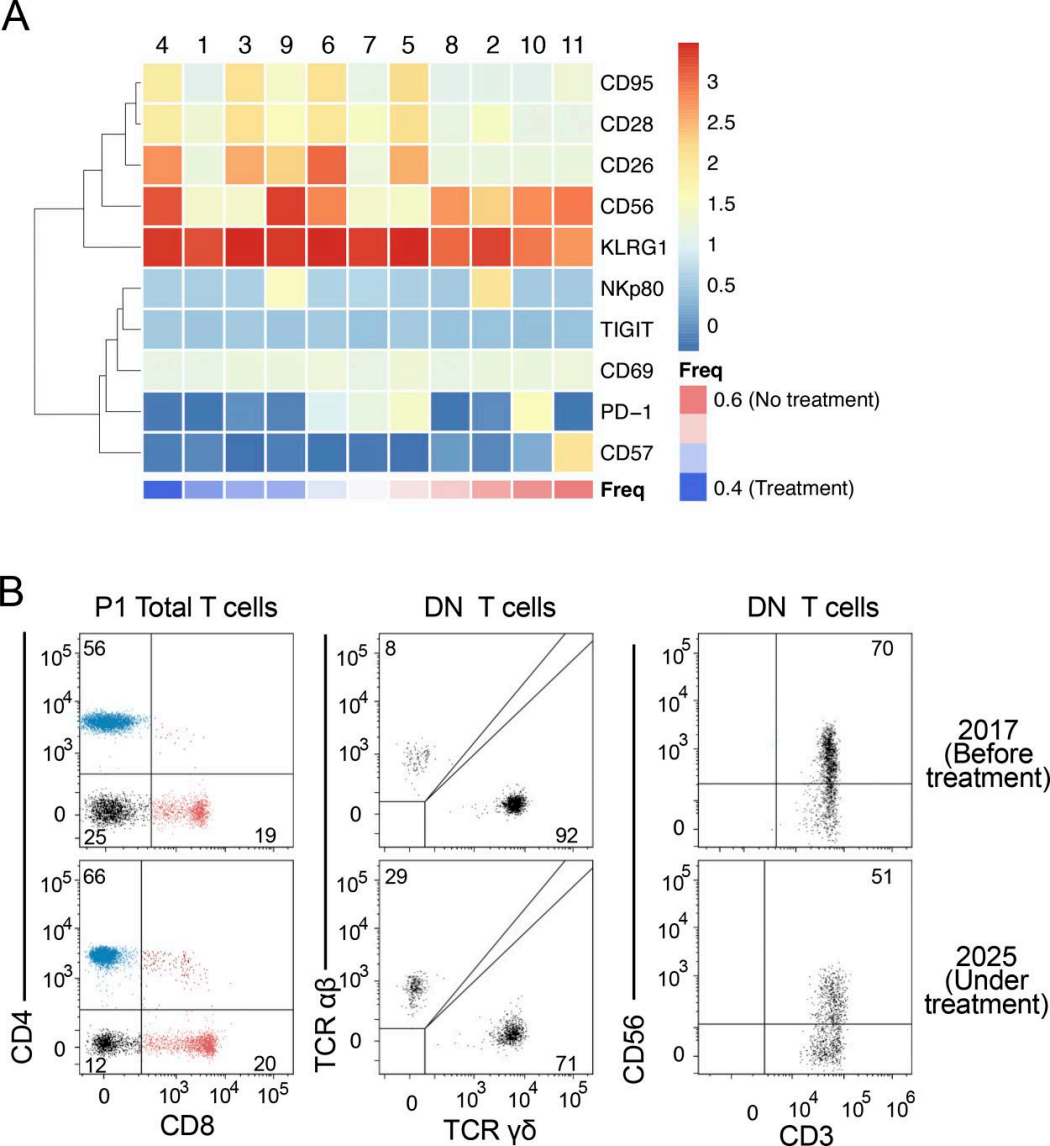
**Heatmap of** **t-SNE** **clusters from P1-derived TCR Vδ2 cells before and under baricitinib treatment. (A)** Heatmap of clustering characterization of TCR Vδ2 cells from P1 before and under treatment with JAK inhibitor according to the expression of the CD95, CD28, CD26, CD56, KLRG1, NKp80, TIGIT, PD-1, and CD57. **(B)** Diagnostic laboratory-derived hemato-immunologic flow cytometry plots of T cells from P1 collected in 2017 (before baricitinib treatment) or in 2025 (under baricitinib treatment). Plots representing CD4 and CD8 expression on total T cells (left), followed by TCR γδ vs. TCR αβ (middle) and CD3 vs. CD56 (right) expression gated on CD4^−^CD8 DN cells. DN, double negative.

Blocking the JAK–STAT signaling pathway with baricitinib did not immediately (within the first 6 mo) alter the imbalance of circulating TCR αβ vs. TCR γδ cells in P1 (Fig. 6 C). The baricitinib treatment did not affect STAT5 phosphorylation in P1-derived TCR γδ cells when exposed to IL-2 in vitro (Fig. 6 D). However, TCR αβ cells from P1 collected during treatment showed lower percentages of pSTAT5 after 4 h of in vitro IL-2 stimulation (Fig. 6 D). There were no differences in the phenotype of TCR Vδ2 cells before and after baricitinib treatment. The marker clusters, including CD95, CD28, CD26, CD56, KLRG1, NKp80, TIGIT, CD69, PD-1, and CD57, were very similar (Fig. 6 E). Additionally, the cluster frequency variation was low before and after treatment (Fig. S5 A). However, a relative decrease in total TCR γδ and TCR Vδ2 cells, associated with a relative increase in TCR αβ cells, was observed over the years under baricitinib treatment (Table 1; and Fig. 6, F and G), with levels currently approaching those seen in healthy donors (Table 1, Fig. 1 D, and Fig. S5 B). While we have not performed NGS-based *STAT5B* sequencing of sorted TCR γδ cells from P1 in the current sample under baricitinib treatment, Sanger sequencing of TCR γδ cell–derived cDNA as a semiquantitative readout revealed the STAT5 GOF mutation to be near the detection limit (Fig. 6 H). NGS-based TCRγ sequencing of a current (2024) sample under baricitinib treatment reveals persistence of the top clone detected in 2017, while the second most abundant clone from 2017 is now ranked fourth. The top 3 clone from 2017 is now ranked fifth (Table 3). Thus, while the relative (and absolute) TCR γδ cells normalized under baricitinib, the spectrum of TCR clones remains stable over time.

## Discussion

Persistent STAT5 activation caused by somatic mutations has been described to favor leukemogenesis and the growth of several tumors (14, 59). Somatic STAT5 GOF mutations in T cells plus myeloid cells have been described to drive early-onset eosinophilia, urticaria, and dermatitis, T cell lymphocytosis, and lymphoproliferation (60, 61). Additionally, STAT5 GOF mutations are found in myeloid neoplasia in elderly patients, often in conjunction with other somatic mutations (62). GOF STAT5B mutations may drive both primary- or lymphocyte-variant hypereosinophilia (63). Here, we present a detailed, long-term (16-year follow-up) study of a chronic immune disorder driven by a TCR γδ cell–specific STAT5B p.Y665F GOF mutation, associated with a persistent, stable increase in peripheral TCR γδ cells.

How does the observed polyclonal γδ T cell expansion in P1 relate to T-LGL leukemia? T-LGL expansions have a broad spectrum, with T cell clonality of uncertain significance (T-CUS) representing the physiologic/indolent side, and aggressive T-LGL leukemia representing the life-threatening counterpart (24). T-LGL leukemia is classically associated with peripheral lymphocytosis (>2 G/L) caused by clonal expansion of CD8^+^ TCR αβ cells, which often carry STAT3 GOF mutations (64). Notably, classical T-LGL leukemia usually affects older adults, whereas our index patient and other reported pediatric cases of T-LGL expansions (65) were identified at a young age. TCR γδ T-LGL leukemia accounts for only 15% of all T-LGL leukemia cases (66), and therein represents a subset of more severe disease with lower survival rates (67). T-LGL leukemia with STAT5B mutations have been identified in only 2% of TCR αβ T-LGL leukemia patients, the p.N642H mutation being associated with faster leukemic progression (26). One rare form of T-LGL leukemia, characterized by clonal expansion of CD4^+^ TCR αβ cells, is typically associated with STAT5 GOF mutations (68).

T cell clonality is a hallmark of T-LGL leukemia; however, normal adaptive immune function also involves clonal T cell expansion in response to antigens. Thus, interpretation of T cell clonality is not trivial. A recent study analyzing NGS-based human TCR γδ sequencing across the lifespan and tissues demonstrates clonal expansions in adults as the rule rather than the exception (69). Flow cytometry analysis allows only the determination of the TCR Vγ and Vδ chains but is not suitable to dissect the clonality of TCR γδ expansions. The patients analyzed here had normal absolute and relative total T cell counts, an elevated proportion being TCR γδ–positive and CD4-negative. γδ TCR sequencing revealed top clone frequencies between 5 and 15% with <10-fold increase compared with the background, arguing against conspicuous clonality (70). Thus, while there is no black/white distinction between T-CUS and indolent T-LGL leukemia, our patients would be classified within the former. In keeping, unlike typical T-LGL leukemia patients, P1 and all other patients studied here did not display absolute lymphocytosis, anemia, or neutropenia, which are red flags for a hematology referral. This indicates that many patients with similar molecular pathogenesis may remain undiagnosed or receive alternative diagnoses, such as unspecified connective tissue disease or chronic fatigue syndrome. P1, in a remarkable 16-year follow-up, has not yet experienced the usual complications associated with T-LGL leukemia, such as overt autoimmune conditions like rheumatoid arthritis or pure red cell aplasia. However, P1’s quality of life was significantly reduced due to recurring, though usually mild, respiratory tract infections, extensive erosive stomatitis, joint pain in the large joints (shoulders and knees), and chronic fatigue. His systemic hyperinflammatory condition was characterized by neutrophilia (in the absence of smoking), monocytosis, mild eosinophilia, and increased CD21^low^ B cells, which have recently been shown to expand in response to IFN-γ (71). Notably, the administration of JAK inhibitors is currently uncommon in T-LGL leukemia. Indeed, none of the 137 TCR γδ T-LGL leukemia cases in a recent multicenter analysis received this treatment (67). Instead, methotrexate, cyclophosphamide, and cyclosporine are the main medications used for patients with T-LGL leukemia who have cytopenia or autoimmune conditions. Among the 137 patients with TCR γδ T-LGL leukemia, cyclosporine appeared to be more effective than methotrexate in those with cytopenias (67). In contrast, JAK inhibition has been successfully used in two pediatric patients with severe hypereosinophilia caused by somatic STAT5B GOF mutations in both myeloid and lymphoid lineages (56). In one of these patients, JAK inhibition was complicated by *Mycobacterium avium* osteomyelitis (72). Very recently, Schmitt et al. described a pediatric case of mosaic p.T628S STAT5B GOF with central nervous system (CNS) demyelinating inflammation, eosinophilia, and lymphocytosis treated with the JAK inhibitor tofacitinib, which was associated with clinical improvement (73). In contrast to P1, the STAT5B GOF mutation was found in ∼50% of reads in all tested myeloid and lymphocyte subsets. Immunophenotypically, mildly increased IgE and IgM were shared with P1, while lymphocytosis and eosinophilia were much more prominent in comparison with P1. P1 also had less acute and systemic disease, as he lacked CNS inflammation and lymphoproliferation, which ultimately required a bone marrow transplant in the p.T628S STAT5B GOF case (73). In P1, JAK inhibition improved reported susceptibility to infection, erosive stomatitis, and most inflammation-related blood biomarkers. However, chronic fatigue and arthralgia remained essentially unchanged. Our findings showed a slow but steady decrease in TCR γδ cells under JAK inhibition, aligning with the expected reduction in STAT5-driven hyperproliferation. We have not yet observed any severe or opportunistic infections in P1 under JAK inhibition. The patient had received extensive vaccinations in accordance with national guidelines for immunosuppressed patients. Besides JAK inhibition, also targeted mTOR inhibition has also been successfully used to control lymphoproliferation in patients with somatic STAT5B mutations (61).

Our understanding of the molecular biology underlying T-LGL expansions remains limited. We could not find any studies that have explored human TCR γδ T-LGL cell activation in response to pAgs. A recent study performed WES and analyzed transcriptomic data from bulk PBMC-derived DNA and RNA in 105 patients with T-LGL leukemia, including 12 with TCR γδ T-LGL leukemia. The study focused on comparing STAT3-mutated vs*.* STAT3-wild-type T-LGL leukemia samples (74). That study revealed upregulation of interferon signaling pathways, supporting the use of JAK inhibitors in T-LGL leukemia. Only one previous study has characterized transcriptomes in TCR γδ T-LGL leukemia vs. nonleukemic TCR γδ subsets (44), without examining the presence or absence of somatic STAT3 or STAT5 mutations. The study demonstrated that the expression profiles of TCR γδ T-LGL leukemia most closely resembled those of the TEMRA TCR γδ subset in HCs. The published transcriptomic T-LGL leukemia profile did not match that of P1’s TCR γδ cells. The *IFNG* gene, which encodes IFN-γ, was highly upregulated, and the expression of pro-apoptotic genes was lower in TCR γδ T-LGL leukemia cells than in HCs (44).

We have examined the biological implications of STAT5 GOF in TCR Vδ2 cells using three approaches. First, flow cytometry showed that P1-derived TCR Vδ2 cells expressed a more innate-like phenotype and had enhanced effector functions compared with Vδ2 cells from HCs. This was evident from the increased expression of CD26, CD56, and NKp80 in P1 cells (75, 76, 77).

Second, we studied how TCR Vδ2 cells respond to the prototypic pAg HMBPP. T cells exposed to HMBPP exhibited stronger TCR-mediated proliferation of TCR Vδ2 cells from P1 vs. HCs. Also, ex vivo analysis of these cells showed increased CD57 expression in HCs compared with P1. In CD4^+^ and CD8^+^ T cells, CD57-positive cells are more differentiated (27) and have impaired proliferative capacity (27, 78, 79). This is consistent with the higher proliferative potential of P1 cells, which express lower levels of CD57 than HCs. The differences in proliferation were observed under suboptimal T cell stimulation, whereas cells from healthy donors can proliferate robustly when challenged with a strong stimulus, such as PHA. Both observations support that the STAT5 GOF mutation is linked to enhanced cell proliferation, in keeping with the relative expansion of TCR Vδ2 cells in P1’s peripheral blood. The gene transcription analysis of P1’s TCR γδ cells consistently revealed higher expression of genes associated with survival and expansion (*NT5E*, *COL6A1*, *CDKN1C*) (30, 31, 32). Genes that control cell growth were also found to be overexpressed in P1 (*COL6A1*, *CDCP1*, *SH3BGRL2*, *SOCS1*, and *SOCS2*) (34, 35, 36, 80). This suggests an intact negative feedback mechanism counteracting the STAT5 GOF–dependent persistent T cell expansion, eventually precluding progression to T-LGL leukemia.

At the transcriptional level, TCR γδ cells from P1 showed changes in IL-2 signaling, as well as in genes related to Th17 and Th9 differentiation. STAT5B GOF TCR γδ cells showed decreased expression of Th17 signature genes, including *RORC*, *MAF*, *LEF1*, *IL17RE*, *KIT*, *IL23R*, *CCR6*, *IL12RB1*, and *ADAM12*. This resembles the transcriptional changes observed in TCR γδ cells from c-MAF knockout mice, which show a block in IL-17 differentiation during early thymocyte development (81). Lower expression of the *MAF*, *RORC*, and *CCR6* genes might result from ongoing STAT5 activity. STAT5 directly targets and suppresses transcription of these genes (82, 83, 84, 85, 86), thus blocking Th17 differentiation.

Activation of TCR Vδ2 cells with HMBPP did not show any differences in the levels of Th1-, Th2-, Th9-, or Th17-related cytokines. However, stimulation with PMA/ionomycin led to a higher overall accumulation of Th1 cytokines, accompanied by a subtle accumulation of Th2 cytokines and IL-9 in TCR Vδ2 cells from P1 compared with HCs, consistent with the changes observed at the transcriptional level. No Th17 cytokines were detected in this set of experiments.

The unstimulated or IL-2–stimulated condition from the RNA-seq experiment does not necessarily reveal the full cytokine potential of the T cells (87, 88). In contrast, PMA/ionomycin stimulation used in the T cell lines induces a supramaximal signaling that exposes the potential of cytokine production of T cells, showing differences that might not be visible in untreated or IL-2–treated conditions (89). Recently, a model has been proposed for Th17 commitment in TCR γδ cells. This model suggests that the transcription factor SOX13 activates c-MAF, which in turn activates RORγ (51). Based on this model and the observed transcriptome of P1, the STAT5 GOF might suppress MAF and RORC expression, as seen in TCR γδ cells from c-MAF knockout mice (81). Notably, STAT5 plays a crucial role in Th2 differentiation. Although it does not enhance the expression of the Th2-related gene GATA3, it does activate the expression of the IL-4 gene (90). We observed higher levels of both intracellular and secreted IL-4 and IL-13 in TCR Vδ2 cell lines from P1 patients than in those from HCs.

Additionally, TCR Vδ2 cells from P1 patients exhibited higher intracellular IL-9 levels than those from HCs. Overall, the transcriptomic analysis revealed STAT5-related changes in gene expression, including suppression of Th17 signature genes, and imbalances among the Th1, Th2, and Th9 cytokine signatures. A limitation of our study is that we analyzed bulk TCR γδ cells and thus cannot functionally discriminate between *STAT5B*-mutated vs.* STAT5B*-wild-type cells at the single-cell level.

It remains unclear whether an underlying cause triggered the acquisition and/or maintenance of STAT5B GOF. Chronic viral infections caused by CMV, EBV, and HIV are suspected to contribute to the context of T-LGL leukemia (91, 92). However, there was neither clinical nor laboratory evidence of a chronic viral infection, and the EBV PCR in peripheral blood was consistently negative, even under JAK inhibition. WES in P1 identified two additional germline mutations in *TYK2*, one with a CADD score of 33 in a highly conserved region, together with another synonymous mutation (Fig. 1 D and Table 4). TYK2 loss-of-function resulting from compound-heterozygous or homozygous mutations has a distinct unrelated clinical phenotype (93). Germline-activating *TYK2 *mutations have been identified as a predisposing factor to the development of acute lymphoblastic leukemia in children (94). Therefore, it is possible that dysregulated TYK2 activity contributed to the development and/or maintenance of STAT5B GOF–mutated TCR γδ cells, experimental analysis of which is beyond the scope of this manuscript. A very recent comprehensive study of T-LGL leukemia patients has revealed an unexpectedly high co-occurrence of rare, damaging germline variants in genes linked to IEI (95).

In summary, a patient with a chronic hyperinflammatory immune dysregulation carried a TCR γδ cell–specific STAT5B GOF mutation despite normal peripheral lymphocyte counts. Our search within our own multicenter prospective cohort of patients with immune dysregulation reveals several other patients with a similar clinical and immunophenotypic profile. In all those patients, TCR sequencing revealed polyclonal T cell populations. The culprit immunophenotypic anomaly (i.e., an altered TCR αβ vs. TCR γδ ratio) in such patients may remain unnoticed. We advise that individuals with chronic immune dysregulation, normal absolute lymphocyte counts and a higher proportion of CD4^−^CD8^−^ T cells should be analyzed for relative TCR γδ cell expansion. Among such patients, we identify an individual with a TCR γδ cell–specific STAT5B GOF mutation, suitable for targeted treatment with JAK inhibitors. We have not yet performed a thorough molecular dissection of all other individuals identified with chronically altered TCR αβ vs. TCR γδ ratios listed in Table 1. In one patient (cohort number 86), WES analysis of gDNA from sorted TCR γδ cells detected neither STAT3 nor STAT5 GOF mutations that could have explained the expansion of TCR γδ cells. However, this individual’s TCR Vδ2 cells, comprising the majority of his TCR γδ cells, showed high reactivity to MR1, an MHC class I–like molecule, suggesting an antigen-driven expansion of these cells (96). Besides STAT5-dependent and antigen-driven expansion, TCR γδ cell generation, maintenance, and function are affected by numerous other IEI genes and associated signaling pathways, as recently summarized by Sagar and Ehl (97).

Within a cohort of patients with chronic susceptibility to infection, a subset exhibits a persistently imbalanced TCR αβ vs. TCR γδ ratio as the primary phenotypic anomaly, which may be easily overlooked in standard immunologic assessments. Underlying molecular reasons in such patients include TCR γδ–specific STAT5B GOF, which may be addressed by tailored immune modulation.

## Materials and methods

### Prospective cohort of patients with immune dysregulation

The cohort and associated immune cell characterization have been approved by the Ethical Commission of Northwestern and Central Switzerland (EKNZ-2015-187).

An experienced clinical immunologist clinically evaluates patients, and only those with likely primary immune dysregulation (i.e., excluding patients with secondary immune dysregulation) are enrolled.

There is written consent for the use of the patient-derived CT-based imaging and images.

### Sex and age as a biological variable

Sex and age were taken as biological variables. HCs and P1 were sex- and age-matched for sample collection.

### Cell isolation

PBMCs and granulocytes were isolated using standard density-gradient centrifugation (Lymphoprep, Axis-Shield). To isolate granulocytes, the erythrocyte/granulocyte fraction was lysed with RBC lysis buffer (#158103; Qiagen). Monocytes and eosinophils were then isolated using a negative selection kit from Stemcell, following the manufacturer’s instructions.

### Cell lines

THP-1 is a human monocytic leukemia cell line obtained from the American Type Culture Collection (No. TIB-202). The tumor cells were grown in RPMI 1640 medium with 10% heat-inactivated fetal calf serum (FCS), 1 mM sodium pyruvate, 1× nonessential amino acids, 1× stable glutamine, and 50 µg/ml kanamycin at 37°C in 5% CO_2_. All reagents were purchased from BioConcept. We regularly checked that the cell lines were free of *Mycoplasma* contamination.

TCR Vγ9Vδ2 cell lines were sorted by flow cytometry from PBMCs using anti-Vδ2 and anti-Vγ9 mAbs. T cell lines were then cultured and expanded in the presence of 100 U/ml of human recombinant IL-2 (#200-02; PeproTech), 1 µg/ml of PHA (#30852801 HA16; Remel), and irradiated PBMCs (5 × 10^5^ cells/ml).

### T cell proliferation assays

For proliferation assays, PBMCs were labeled with carboxyfluorescein succinimidyl ester (#C34554; Thermo Fisher Scientific) or CellTrace Violet (#C34557; Thermo Fisher Scientific) according to the manufacturer’s instructions. The labeled cells were then stimulated with 0.4 nM HMBPP (#95098; Sigma-Aldrich) or 1 µg/ml PHA (#R30852701, Thermo Fisher Scientific). Proliferation and the upregulation of CD69, CD137, CD39, and ICOS were assessed by flow cytometry on day 3.5 after stimulation.

### T cell activation assays

For the activation assay, THP-1 cells were preincubated with different doses of HMBPP for 3 h at 37°C, then cocultured with TCR Vγ9Vδ2 cell lines from either HCs or P1 for 18 h at 37°C and 5% CO_2_, at an effector-to-target ratio of 1:2. Activation was measured using ELISA.

### ELISA

Release of IFN-γ, TNF-α, IL-4, and IL-13 was measured using ELISA, as previously described (98). The methods used were human IFN-γ (capture MD-1 mAb [#507502]; revealing biotinylated 4S.B3 mAb [#502504]; BioLegend); human TNF-α (capture Mab1 mAb [#502802]; revealing biotinylated Mab11 mAb [#502904]; BioLegend); human IL-4 (capture 8D4-8 mAb [#500702]; revealing biotinylated MP4-25D2 mAb [#biotinylated MP4-25D2]; BioLegend); and human IL-13 (capture JES10-5A2 rat Ab [#10125-01]; revealing biotinylated SB126d Rat Ab [#15930-08]; SouthernBiotech).

### Flow cytometry

Clinical routine immunophenotyping was performed as previously described (99) (see Table 2). Clinical routine hemato-immunologic phenotyping, including T-LGL–related markers, was performed as previously described (100) (see Table 1).

Antibodies used for research-based flow cytometry, including clones, are listed in Table 5.

**Table 5.** List of antibodies used for flow cytometry-based immune-phenotyping

Conjugated mAbs specific for human surface markers				
Target	Clone	Fluorochrome	Provider	Catalog number
TCR γδ	B1	PE	BioLegend	361108
TCR γδ	11F2	PE	BD Biosciences	340887
TCR γδ	11F2	BUV395	BD Biosciences	745681
CD3ε	SK7	PerCP-Cy5.5	BioLegend	344807
CD3ε	UCHT1	APC	BioLegend	300411
CD3ε	UCHT1	APC	BioLegend	300411
CD3ε	UCHT1	AF488	BioLegend	300454
TCR αβ	IP26	PE	BioLegend	984702
TCR γδ	B1	PE-Dazzle 594	BioLegend	331225
TCR Vγ9	B3	PerCP-Cy5.5	BioLegend	331321
TCR Vδ1	TS-1	FITC	Thermo Fisher Scientific	TCR2055
TCR Vδ2	B6	BV711	BioLegend	331412
TCR Vδ2	B6	FITC	BioLegend	331405
TCR Vδ2	B6	APC	BioLegend	331418
CD4	OKT4	AF700	BioLegend	317425
CD8	RPA-T8	BUV496	BD Biosciences	612442
ICOS	DX29	BUV661	BD Biosciences	741664
CD39	A1	BV711	BioLegend	328228
CD137	4B4-1	PE-Cy7	BioLegend	309818
CD137	4B4-1	APC	BioLegend	309810
CD137	4B4-1	APC-Cy7	BioLegend	309830
CD69	FN50	BV605	BioLegend	310938
CD69	FN50	PE	BioLegend	310906
CD69	FN50	PE-Cy7	BioLegend	310912
CD57	HNK-1	PerCP-Cy5.5	BioLegend	359622
CD28	CD28.2	AF647	BioLegend	302954
CD14	63D3	APC-Cy7	BioLegend	367108
CD19	H1B19	APC-Cy7	BioLegend	302218
CD26	M-A261	BV480	BD Biosciences	746696
KLRG1	2F1/KLRG1	APC	BioLegend	138412
CD95	DX2	BV650	BioLegend	305642
CD161	DX12	BUV737	BD Biosciences	748948
CD56	5.1H11	BV785	BioLegend	362550
NKp80	5D12	PE	BioLegend	346706
PD-1	EH12.2H7	BV421	BioLegend	329920
TIGIT	741182	BUV395	BD Biosciences	741182
CCR7	3D12	BUV563	BD Biosciences	741317
CD45RA	5H9	BUV661	BD Biosciences	741654

Conjugated mAbs specific for human intracellular cytokines				
Target	Clone	Fluorochrome	Provider	Catalog number
IL-2	5344.111	BV650	BD Biosciences	563467
IL-4	MP4-25D2	BV510	BioLegend	500836
IL-10	JES3-9D7	PE-Cy7	BioLegend	501420
IL-13	JES10-5A2	PE	BioLegend	501903
IL-17A	BL168	AF488	BioLegend	512308
GM-CSF	BVD2-21C11	Pacific Blue	BioLegend	502314
IL-9	MH9D1	PerCP-eFluor 710	Thermo Fisher Scientific	46-7098-42
IL-22	2G12A41	PE	BioLegend	366703
IFN-γ	4S.B3	BUV737	BD Biosciences	612845
TNF-α	MAb11	BV785	BioLegend	502948
pSTAT5	47/Stat5(pY694)	AF488	BD Pharmingen	612598
Isotype mouse IgG1	MOPC-21	AF488	BD Pharmingen	557702

### Surface staining

Cells were stained with Zombie NIR (#423106; BioLegend) or LIVE/DEAD BLUE (#L23105; Thermo Fisher Scientific) fixable viability dyes in PBS for 20 min at 4°C, then surface-stained. Anti-human mAbs were used to stain cells in FACS buffer (PBS, 0.5% BSA, 0.02% NaN3) at 4°C for 20 min. Viability was assessed by adding DAPI to PBS after cell surface staining. Cells were acquired using an Aurora spectral analyzer (Cytek) or a CytoFLEX flow cytometer (Beckman Coulter) and analyzed with FlowJo v10 software (LLC). Cell sorting was performed by flow cytometry on a BD Influx, a BD Aria III, or a FACSMelody Cell Sorter (BD) cytometer.

### Intracellular staining

Staining of pSTAT5 (Y694 STAT5A/Y699 STAT5B) was done on sorted TCR γδ and TCR αβ cells that had been rested overnight in medium at 37°C. The medium used, called complete medium, was RPMI (Sigma-Aldrich), supplemented with 100 U/ml of penicillin–streptomycin (Gibco), 1× MEM nonessential amino acid solution (Gibco), 1× GlutaMAX (Gibco), and 10% FCS (Gibco). The next day, 2 × 10^5^ cells were either stimulated with hIL-2 (300 U/ml, Novartis) for 30 min or left unstimulated at a concentration of 2 × 10^5^ cells/ml. The pSTAT5 antibody from BD Pharmingen, used for flow cytometry, was recently used to characterize STAT5B GOF patients (60). Staining of intracellular cytokines was performed in TCR Vδ2 cell lines treated with 2 µM monensin (#420701; BioLegend) and 20 µg/ml brefeldin A (#420601; BioLegend). The cells were then either stimulated or not with 50 ng/ml PMA (Sigma-Aldrich) and 500 ng/ml ionomycin (Sigma-Aldrich).

Cells were fixed using Fix/Perm buffer (#554714; BD Biosciences) in the dark at room temperature for 20 min. Next, cells were washed in PBS with 2% FCS and then permeabilized with Perm III Buffer (#558050; BD Biosciences) at 4°C for 20 min.

For STAT5 staining, the conjugated antibody and isotype control were incubated at room temperature for 45 min, followed by a 45-min incubation with the secondary antibody at the same temperature.

For intracellular cytokine staining, cells were incubated with cytokine-specific antibodies for 30 min at 4°C. We collected cells using either a CytoFLEX (Beckman Coulter) or an Aurora spectral analyzer (Cytek). Data were analyzed with FlowJo v10 software (FlowJo LLC).

### Endpoint PCR and Sanger sequencing

Endpoint PCR was performed using genomic DNA or cDNA with GoTaq Polymerase (#M7841; Promega) following the manufacturer’s instructions. The primer concentration was 0.5 µM. For cloning the PCR product, Phusion Polymerase (#M0530S; NEB) and the Zero Blunt TOPO PCR cloning kit (#K287540; Thermo Fisher Scientific) were used.

PCR was performed on T Professional TRIO PCR Thermocycler (Core Life Sciences). The PCR products were separated and visualized using a 1.5% agarose gel. Specific bands were then excised and purified using the Qiagen Gel Extraction kit (#28704; Qiagen). Sanger sequencing was conducted by Microsynth.

Primers for the STAT5B Y665F mutation on the cDNA level:

Fw: 5′-AGG​GAG​AAT​TTA​CCA​GGA​CGG-3′

Rev: 5′-TCC​ATG​TAC​GTG​GCG​CTG-3′.

Primers for STAT5B Y665F mutation on the genomic DNA level:

Fw: 5′-AAA​TGG​AGA​TTT​CTA​TTG​GAG​CCA​T-3′

Rev: 5′-TAG​CAG​ACT​CGC​AGG​GAA​CT-3′.

### WES

WES was performed using genomic DNA from CD3^+^TCR γδ^+^ and CD3^+^TCR αβ^+^ cells sorted by flow cytometry ex vivo.

WES has been performed as recently described (101). The presence of rare mutations linked to immunodeficiencies was investigated using a compiled list of primary immunodeficiencies (102). The genome viewer confirmed all reported variants. Immunodeficiency-related gene variants were listed when the CADD score was >15, and the minor allele frequency listed in gnomAD (https://gnomad.broadinstitute.org) was <0.01.

### NGS-based TCR sequencing

Blood or PBMC-derived DNA was used for amplification for TCRγ- or TCRβ-specific CDR3 regions (75–100 base pairs) using Oncomine TCR Pan-Clonality Assay. Data analysis was performed using Torrent Suite and Ion Reporter software.

### RNA isolation and RNA-seq

Cells were lysed in TRIzol reagent (#15596026; Thermo Fisher Scientific), and then, RNA was purified using the Direct-zol RNA kit (#R2061; Zymo Research). The RNA concentration was measured with the Qubit fluorometer (Thermo Fisher Scientific). Library preparation was done at the University of Basel by the Genomic Core Facility. Sequencing was performed on an Illumina NovaSeq S1, yielding 48–60 million reads per sample.

Reads were aligned to the human genome (UCSC version hg38 analysis set) using STAR (version 2.7.0c) with additional options “outFilterMultimapNmax10-- outSAMmultNmax 1.” This allowed multimapping reads to be included, with a maximum of 10 locations and a random selection of one location. The output was then merged across flow cells, sorted, and indexed using SAMtools (version 1.9). We evaluated read and alignment quality using the qQCReport function from the QuasR Bioconductor package (R version 3.5.0, Bioconductor version 3.8). To count the number of reads (5′ ends) overlapping with each gene’s exons, we used the featureCounts function from the subread R/Bioconductor package, assuming an exon union model. Gene annotation was based on Ensembl version 94.

### RNA isolation and cDNA production

Cells were lysed using TRIzol reagent (#15596026; Thermo Fisher Scientific). We then isolated RNA using chloroform (#319988; Sigma-Aldrich), followed by 15-min centrifugation at 4°C and 14,000 *g*, according to the manufacturer’s instructions, and purified it with QIAamp RNA Blood Mini Kit (#52304; Qiagen). To determine the RNA concentration, we used the NanoDrop 2000c (Thermo Fisher Scientific). For DNA digestion, we used the DNase I Amplification–Grade Kit (#18068015; Sigma-Aldrich). Concentrations of 100 ng/μl to 1 μg/μl RNA were used for cDNA synthesis. We annealed random primers (#C1181; Promega) at 70°C for 5 min. cDNA synthesis was done according to the Qiagen GoScript Reverse Transcription System protocol in T Professional TRIO PCR Thermocycler (Core Life Sciences).

### Bioinformatics analysis

Flow cytometry data were exported from FlowJo v10.7.1 and imported into R v4.2.0 using the Bioconductor package flowCore v2.10.0 (103) to perform clustering analysis. Marker expression levels were transformed using the inverse hyperbolic sine transformation (asinh function in R) with a cofactor of 150. Clusters were then computed using R’s implementation of PhenoGraph (104), based on the transformed expression values for nine markers (CD69, CD26, CD28, CD56, CD57, CD161, NKp80, CD95, and PD-1) and a k value of 100. A heatmap showing the average expression of each marker in each cluster was produced using pheatmap v1.0.12 (105), and the clusters were visualized as colors overlaid on a Uniform Manifold Approximation and Projection (UMAP) using the R implementation of UMAP (v0.2.10.0) (106, *Preprint*).

### Statistical analysis and data visualization

Statistical analysis and data visualization were performed using GraphPad Prism (GraphPad Software, Inc.). P values are indicated in the figure panels and legends.

The assembling of figure panels was done using Affinity Designer 2.

### Online supplemental material

The following supplemental materials are provided for this manuscript. Fig. S1 depicts immune cell populations and imaging of P1, as well as STAT5B sequencing in enriched myeloid cell populations. Fig. S2 characterizes the immunophenotype of TCR Vδ2–negative cells from P1. Fig. S3 illustrates proliferation and activation assays of TCR Vδ2 cells challenged with HMBPP in P1 vs. HCs. Fig. S4 compares gene expression in TCR γδ cells and cytokine profiling in P1 vs. HCs. Fig. S5 characterizes P1-derived TCR Vδ2 cells before and under baricitinib treatment.

## Supplementary Material

SourceData FS1 is the source file for Fig. S1.

## Data Availability

The datasets used in this study are not publicly available to protect participant/patient anonymity. Requests to access the datasets can be made to the corresponding author. The disease-associated somatic c.A1994T STAT5B mutation has been submitted to ClinVar (accession number: SCV007540363).

## References

[1] Herman, K.E., and K.L. Tuttle. 2024. Overview of secondary immunodeficiency. Allergy Asthma Proc. 45:347–354. 10.2500/aap.2024.45.240063 39294908

[2] Kim, V.H.D., J.E.M. Upton, B. Derfalvi, K.J. Hildebrand, and C. McCusker. 2025. Inborn errors of immunity (primary immunodeficiencies). Allergy Asthma Clin. Immunol. 20:76. 10.1186/s13223-024-00938-z 39780212 PMC11714877

[3] Cooper, M.A. 2025. Somatic mosaicism in genetic errors of immunity. J. Allergy Clin. Immunol. 155:759–767. 10.1016/j.jaci.2024.11.038 39724970 PMC12020649

[4] Soler-Palacin, P., J.G. Riviere, S.O. Burns, and N.L. Rider. 2025. New tools for diagnosis of primary immunodeficiencies: From awareness to artificial intelligence. Front. Immunol. 16:1593897. 10.3389/fimmu.2025.1593897 40709193 PMC12286987

[5] Kornfeld, J.W., F. Grebien, M.A. Kerenyi, K. Friedbichler, B. Kovacic, B. Zankl, A. Hoelbl, H. Nivarti, H. Beug, V. Sexl, . 2008. The different functions of Stat5 and chromatin alteration through Stat5 proteins. Front. Biosci. 13:6237–6254. 10.2741/3151 18508657 PMC2976847

[6] Cella, N., B. Groner, and N.E. Hynes. 1998. Characterization of Stat5a and Stat5b homodimers and heterodimers and their association with the glucocortiocoid receptor in mammary cells. Mol. Cell. Biol. 18:1783–1792. 10.1128/MCB.18.4.1783 9528750 PMC121408

[7] Hu, X., J. Li, M. Fu, X. Zhao, and W. Wang. 2021. The JAK/STAT signaling pathway: From bench to clinic. Signal. Transduct Target. Ther. 6:402. 10.1038/s41392-021-00791-1 34824210 PMC8617206

[8] Kofoed, E.M., V. Hwa, B. Little, K.A. Woods, C.K. Buckway, J. Tsubaki, K.L. Pratt, L. Bezrodnik, H. Jasper, A. Tepper, . 2003. Growth hormone insensitivity associated with a STAT5b mutation. N. Engl. J. Med. 349:1139–1147. 10.1056/NEJMoa022926 13679528

[9] Klammt, J., D. Neumann, E.F. Gevers, S.F. Andrew, I.D. Schwartz, D. Rockstroh, R. Colombo, M.A. Sanchez, D. Vokurkova, J. Kowalczyk, . 2018. Dominant-negative STAT5B mutations cause growth hormone insensitivity with short stature and mild immune dysregulation. Nat. Commun. 9:2105. 10.1038/s41467-018-04521-0 29844444 PMC5974024

[10] Kasap, N., K. Aslan, L.T. Karakurt, H. Bozkurt, H. Canatan, O. Cavkaytar, A. Eken, and M. Arga. 2022. A novel gain-of-function mutation in STAT5B is associated with treatment-resistant severe atopic dermatitis. Clin. Exp. Allergy. 52:907–910. 10.1111/cea.14148 35426955

[11] Maurer, B., S. Kollmann, J. Pickem, A. Hoelbl-Kovacic, and V. Sexl. 2019. STAT5A and STAT5B-twins with different personalities in hematopoiesis and leukemia. Cancers (Basel). 11:1726. 10.3390/cancers11111726 31690038 PMC6895831

[12] Tremblay, C.S., J. Saw, J.A. Boyle, K. Haigh, V. Litalien, H. McCalmont, K. Evans, R.B. Lock, S.M. Jane, J.J. Haigh, and D.J. Curtis. 2023. STAT5 activation promotes progression and chemotherapy resistance in early T-cell precursor acute lymphoblastic leukemia. Blood. 142:274–289. 10.1182/blood.2022016322 36989489

[13] Kim, U., and H.Y. Shin. 2022. Genomic mutations of the STAT5 transcription factor are associated with human cancer and immune diseases. Int. J. Mol. Sci. 23:11297. 10.3390/ijms231911297 36232600 PMC9569778

[14] Halim, C.E., S. Deng, M.S. Ong, and C.T. Yap. 2020. Involvement of STAT5 in oncogenesis. Biomedicines. 8:316. 10.3390/biomedicines8090316 32872372 PMC7555335

[15] Deseke, M., and I. Prinz. 2020. Ligand recognition by the γδ TCR and discrimination between homeostasis and stress conditions. Cell. Mol. Immunol. 17:914–924. 10.1038/s41423-020-0503-y 32709926 PMC7608190

[16] Hayday, A., J. Dechanet-Merville, J. Rossjohn, and B. Silva-Santos. 2024. Cancer immunotherapy by γδ T cells. Science. 386:eabq7248. 10.1126/science.abq7248 39361750 PMC7616870

[17] Hintz, M., A. Reichenberg, B. Altincicek, U. Bahr, R.M. Gschwind, A.K. Kollas, E. Beck, J. Wiesner, M. Eberl, and H. Jomaa. 2001. Identification of (E)-4-hydroxy-3-methyl-but-2-enyl pyrophosphate as a major activator for human gammadelta T cells in *Escherichia coli*. FEBS Lett. 509:317–322. 10.1016/s0014-5793(01)03191-x 11741609

[18] Vantourout, P., and A. Hayday. 2013. Six-of-the-best: Unique contributions of γδ T cells to immunology. Nat. Rev. Immunol. 13:88–100. 10.1038/nri3384 23348415 PMC3951794

[19] Su, D., M. Shen, X. Li, and L. Sun. 2013. Roles of γδ T cells in the pathogenesis of autoimmune diseases. Clin. Dev. Immunol. 2013:985753. 10.1155/2013/985753 23533458 PMC3600234

[20] Paul, S., Shilpi, and G. Lal. 2015. Role of gamma-delta (γδ) T cells in autoimmunity. J. Leukoc. Biol. 97:259–271. 10.1189/jlb.3RU0914-443R 25502468

[21] Caccamo, N., C. La Mendola, V. Orlando, S. Meraviglia, M. Todaro, G. Stassi, G. Sireci, J.J. Fournié, and F. Dieli. 2011. Differentiation, phenotype, and function of interleukin-17-producing human Vγ9Vδ2 T cells. Blood. 118:129–138. 10.1182/blood-2011-01-331298 21505189

[22] Wesch, D., A. Glatzel, and D. Kabelitz. 2001. Differentiation of resting human peripheral blood gamma delta T cells toward Th1- or Th2-phenotype. Cell. Immunol. 212:110–117. 10.1006/cimm.2001.1850 11748927

[23] Agerholm, R., and V. Bekiaris. 2021. Evolved to protect, designed to destroy: IL-17-producing γδ T cells in infection, inflammation, and cancer. Eur. J. Immunol. 51:2164–2177. 10.1002/eji.202049119 34224140

[24] Semenzato, G., A. Teramo, G. Calabretto, and R. Zambello. 2025. T-cell clones of uncertain significance. When is the rogue clone dangerous? Haematologica. 110:37–46. 10.3324/haematol.2024.286023 39363880 PMC11694120

[25] Yang, R., D.T. Avery, K.J.L. Jackson, M. Ogishi, I. Benhsaien, L. Du, X. Ye, J. Han, J. Rosain, J.N. Peel, . 2022. Human T-bet governs the generation of a distinct subset of CD11c^high^CD21^low^ B cells. Sci. Immunol. 7:eabq3277. 10.1126/sciimmunol.abq3277 35867801 PMC9413977

[26] Rajala, H.L., S. Eldfors, H. Kuusanmäki, A.J. van Adrichem, T. Olson, S. Lagström, E.I. Andersson, A. Jerez, M.J. Clemente, Y. Yan, . 2013. Discovery of somatic STAT5b mutations in large granular lymphocytic leukemia. Blood. 121:4541–4550. 10.1182/blood-2012-12-474577 23596048 PMC3668487

[27] Kared, H., S. Martelli, T.P. Ng, S.L. Pender, and A. Larbi. 2016. CD57 in human natural killer cells and T-lymphocytes. Cancer Immunol. Immunother. 65:441–452. 10.1007/s00262-016-1803-z 26850637 PMC11029668

[28] Bitar, M., A. Boldt, M.-T. Freitag, B. Gruhn, U. Köhl, and U. Sack. 2019. Evaluating STAT5 phosphorylation as a mean to assess T cell proliferation. Front. Immunol. 10:722. 10.3389/fimmu.2019.00722 31024554 PMC6460883

[29] Liberzon, A., C. Birger, H. Thorvaldsdóttir, M. Ghandi, J.P. Mesirov, and P. Tamayo. 2015. The Molecular Signatures Database (MSigDB) hallmark gene set collection. Cell Syst. 1:417–425. 10.1016/j.cels.2015.12.004 26771021 PMC4707969

[30] Fang, F., W. Cao, W. Zhu, N. Lam, L. Li, S. Gaddam, Y. Wang, C. Kim, S. Lambert, H. Zhang, . 2021. The cell-surface 5'-nucleotidase CD73 defines a functional T memory cell subset that declines with age. Cell Rep. 37:109981. 10.1016/j.celrep.2021.109981 34758299 PMC8612175

[31] Fuller, A.M., H.C. Pruitt, Y. Liu, V.M. Irizarry-Negron, H. Pan, H. Song, A. DeVine, R.S. Katti, S. Devalaraja, G.E. Ciotti, . 2024. Oncogene-induced matrix reorganization controls CD8+ T cell function in the soft-tissue sarcoma microenvironment. J. Clin. Invest. 134:e167826. 10.1172/JCI167826 38652549 PMC11142734

[32] Chen, G., K. Subedi, S. Chakraborty, A. Sharov, J. Lu, J. Kim, X. Mi, R. Wersto, M.-H. Sung, and N.-P. Weng. 2018. Ezh2 regulates activation-induced CD8^+^ T cell cycle progression via repressing *Cdkn2a* and *Cdkn1c* expression. Front. Immunol. 9:549. 10.3389/fimmu.2018.00549 29632530 PMC5879148

[33] Gerard, A., R.A. van der Kammen, H. Janssen, S.I. Ellenbroek, and J.G. Collard. 2009. The Rac activator Tiam1 controls efficient T-cell trafficking and route of transendothelial migration. Blood. 113:6138–6147. 10.1182/blood-2008-07-167668 19139083

[34] Briceno, P., E. Rivas-Yañez, M.V. Rosemblatt, B. Parra-Tello, P. Farías, L. Vargas, V. Simon, C. Cárdenas, A. Lladser, F. Salazar-Onfray, . 2021. CD73 ectonucleotidase restrains CD8+ T cell metabolic fitness and anti-tumoral activity. Front. Cell Dev. Biol. 9:638037. 10.3389/fcell.2021.638037 33681221 PMC7930398

[35] Huang, H., Y. Pan, Q. Mai, C. Zhang, Q. Du, Y. Liao, S. Qin, Y. Chen, J. Huang, J. Li, . 2024. Targeting CDCP1 boost CD8+ T cells-mediated cytotoxicity in cervical cancer via the JAK/STAT signaling pathway. J. Immunother. Cancer. 12:e009416. 10.1136/jitc-2024-009416 39455095 PMC11529519

[36] Nie, Z., S. Cai, Z. Wei, Y. Li, L. Bian, C. Wang, and C. Wang. 2021. SH3BGRL2 functions as a crucial tumor suppressor in glioblastoma tumorigenesis. Biochem. Biophys. Res. Commun. 547:148–154. 10.1016/j.bbrc.2021.02.035 33610914

[37] Wang, S., V. Böhnert, A.J. Joseph, V. Sudaryo, G. Skariah, J.T. Swinderman, F.B. Yu, V. Subramanyam, D.M. Wolf, X. Lyu, . 2023. ENPP1 is an innate immune checkpoint of the anticancer cGAMP-STING pathway in breast cancer. Proc. Natl. Acad. Sci. USA. 120:e2313693120. 10.1073/pnas.2313693120 38117852 PMC10756298

[38] Akimova, T., U.H. Beier, L. Wang, M.H. Levine, and W.W. Hancock. 2011. Helios expression is a marker of T cell activation and proliferation. PLoS One. 6:e24226. 10.1371/journal.pone.0024226 21918685 PMC3168881

[39] Chikuma, S. 2017. CTLA-4, an essential immune-checkpoint for T-cell activation. Curr. Top. Microbiol. Immunol. 410:99–126. 10.1007/82_2017_61 28900679

[40] Tan, H., K. Yang, Y. Li, T.I. Shaw, Y. Wang, D.B. Blanco, X. Wang, J.-H. Cho, H. Wang, S. Rankin, . 2017. Integrative proteomics and phosphoproteomics profiling reveals dynamic signaling networks and bioenergetics pathways underlying T cell activation. Immunity. 46:488–503. 10.1016/j.immuni.2017.02.010 28285833 PMC5466820

[41] Harberts, A., C. Schmidt, J. Schmid, D. Reimers, F. Koch-Nolte, H.-W. Mittrücker, and F. Raczkowski. 2021. Interferon regulatory factor 4 controls effector functions of CD8^+^ memory T cells. Proc. Natl. Acad. Sci. USA. 118:e2014553118. 10.1073/pnas.2014553118 33859042 PMC8072204

[42] Starkey, M.R., D.H. Nguyen, A.T. Essilfie, R.Y. Kim, L.M. Hatchwell, A.M. Collison, H. Yagita, P.S. Foster, J.C. Horvat, J. Mattes, and P.M. Hansbro. 2014. Tumor necrosis factor-related apoptosis-inducing ligand translates neonatal respiratory infection into chronic lung disease. Mucosal Immunol. 7:478–488. 10.1038/mi.2013.65 24045576

[43] Man, K., S.S. Gabriel, Y. Liao, R. Gloury, S. Preston, D.C. Henstridge, M. Pellegrini, D. Zehn, F. Berberich-Siebelt, M.A. Febbraio, . 2017. Transcription factor IRF4 promotes CD8^+^ T cell exhaustion and limits the development of memory-like T cells during chronic infection. Immunity. 47:1129–1141.e5. 10.1016/j.immuni.2017.11.021 29246443

[44] Kallemeijn, M.J., D. de Ridder, J. Schilperoord-Vermeulen, M.Y. van der Klift, Y. Sandberg, J.J.M. van Dongen, and A.W. Langerak. 2017. Dysregulated signaling, proliferation and apoptosis impact on the pathogenesis of TCRγδ+ T cell large granular lymphocyte leukemia. PLoS One. 12:e0175670. 10.1371/journal.pone.0175670 28407008 PMC5391076

[45] Narayan, K., K.E. Sylvia, N. Malhotra, C.C. Yin, G. Martens, T. Vallerskog, H. Kornfeld, N. Xiong, N.R. Cohen, M.B. Brenner, . 2012. Intrathymic programming of effector fates in three molecularly distinct γδ T cell subtypes. Nat. Immunol. 13:511–518. 10.1038/ni.2247 22473038 PMC3427768

[46] Huang, L., X. Zhang, J. Fan, X. Liu, S. Luo, D. Cao, Y. Liu, Z. Xia, H. Zhong, C. Chen, . 2023. EGFR promotes the apoptosis of CD4^+^ T lymphocytes through TBK1/Glut1 induced Warburg effect in sepsis. J. Adv. Res. 44:39–51. 10.1016/j.jare.2022.04.010 35618635 PMC9936423

[47] Verdeil, G. 2016. MAF drives CD8^+^ T-cell exhaustion. Oncoimmunology. 5:e1082707. 10.1080/2162402X.2015.1082707 27057466 PMC4801447

[48] Zhou, X., and H.-H. Xue. 2012. Cutting edge: Generation of memory precursors and functional memory CD8+ T cells depends on T cell factor-1 and lymphoid enhancer-binding factor-1. J. Immunol. 189:2722–2726. 10.4049/jimmunol.1201150 22875805 PMC3437003

[49] Eberl, G., and D.R. Littman. 2004. Thymic origin of intestinal alphabeta T cells revealed by fate mapping of RORgammat+ cells. Science. 305:248–251. 10.1126/science.1096472 15247480

[50] Odagiu, L., J. May, S. Boulet, T.A. Baldwin, and N. Labrecque. 2020. Role of the orphan nuclear receptor NR4A family in T-cell biology. Front. Endocrinol. (Lausanne). 11:624122. 10.3389/fendo.2020.624122 33597928 PMC7883379

[51] Sagar, M. Pokrovskii, J.S. Herman, S. Naik, E. Sock, P. Zeis, U. Lausch, M. Wegner, Y. Tanriver, D.R. Littman, . 2020. Deciphering the regulatory landscape of fetal and adult γδ T-cell development at single-cell resolution. EMBO J. 39:e104159. 10.15252/embj.2019104159 32627520 PMC7327493

[52] Napier, R.J., E.J. Lee, M.P. Davey, E.E. Vance, J.M. Furtado, P.E. Snow, K.A. Samson, S.J. Lashley, B.R. Brown, R. Horai, . 2020. T cell-intrinsic role for Nod2 in protection against Th17-mediated uveitis. Nat. Commun. 11:5406. 10.1038/s41467-020-18961-0 33106495 PMC7589501

[53] Agnello, L., A. Masucci, M. Tamburello, R. Vassallo, D. Massa, R.V. Giglio, M. Midiri, C.M. Gambino, and M. Ciaccio. 2025. The role of killer Ig-like receptors in diseases from A to Z. Int. J. Mol. Sci. 26:3242. 10.3390/ijms26073242 40244151 PMC11989319

[54] Harbour, S.N., D.F. DiToro, S.J. Witte, C.L. Zindl, M. Gao, T.R. Schoeb, G.W. Jones, S.A. Jones, R.D. Hatton, and C.T. Weaver. 2020. T_H_17 cells require ongoing classic IL-6 receptor signaling to retain transcriptional and functional identity. Sci. Immunol. 5:eaaw2262. 10.1126/sciimmunol.aaw2262 32680955 PMC7843024

[55] Micossé, C., L. von Meyenn, O. Steck, E. Kipfer, C. Adam, C. Simillion, S.M. Seyed Jafari, P. Olah, N. Yawlkar, D. Simon, . 2019. Human “T_H_9” cells are a subpopulation of PPAR-γ^+^ T_H_2 cells. Sci. Immunol. 4:eaat5943. 10.1126/sciimmunol.aat5943 30658968

[56] Eisenberg, R., M.D. Gans, T.R. Leahy, F. Gothe, C. Perry, M. Raffeld, L. Xi, S. Blackstone, C. Ma, S. Hambleton, and J.D. Milner. 2021. JAK inhibition in early-onset somatic, nonclonal STAT5B gain-of-function disease. J. Allergy Clin. Immunol. Pract. 9:1008–1010.e2. 10.1016/j.jaip.2020.11.050 33290916 PMC9175514

[57] Bilori, B., S. Thota, M.J. Clemente, B. Patel, A. Jerez, M. Afable Ii, and J.P. Maciejewski. 2015. Tofacitinib as a novel salvage therapy for refractory T-cell large granular lymphocytic leukemia. Leukemia. 29:2427–2429. 10.1038/leu.2015.280 26449659

[58] Damsky, W., D. Peterson, J. Ramseier, B. Al-Bawardy, H. Chun, D. Proctor, V. Strand, R.A. Flavell, and B. King. 2021. The emerging role of Janus kinase inhibitors in the treatment of autoimmune and inflammatory diseases. J. Allergy Clin. Immunol. 147:814–826. 10.1016/j.jaci.2020.10.022 33129886

[59] Smith, M.R., L.R.F. Satter, and A. Vargas-Hernandez. 2022. STAT5b: A master regulator of key biological pathways. Front. Immunol. 13:1025373. 10.3389/fimmu.2022.1025373 36755813 PMC9899847

[60] Ma, C.A., L. Xi, B. Cauff, A. DeZure, A.F. Freeman, S. Hambleton, G. Kleiner, T.R. Leahy, M. O’Sullivan, M. Makiya, . 2017. Somatic STAT5b gain-of-function mutations in early onset nonclonal eosinophilia, urticaria, dermatitis, and diarrhea. Blood. 129:650–653. 10.1182/blood-2016-09-737817 27956386 PMC5290989

[61] Grun, S., A. Rensing-Ehl, T. Suske, J. Wolter-Mess, J. Gehrig, J. Mann, C. König, M. Hauri, M. Heeg, T.R. Leahy, . 2025. Somatic STAT5B^N642H^ mutations shape variable immune landscapes resulting in heterogenous immune diseases. J. Allergy Clin. Immunol. 157:964–976. 10.1016/j.jaci.2025.12.993 41453450

[62] Kavesh, M., M. Mohebnasab, M.R. Angel, W. Xie, P.W. Raess, W. Cui, R.D. Press, G. Yang, and P. Li. 2023. Distinguishing STAT3/STAT5B-mutated large granular lymphocyte leukemia from myeloid neoplasms by genetic profiling. Blood Adv. 7:40–45. 10.1182/bloodadvances.2022008192 35939786 PMC9813527

[63] Umrau, K., K. Naganuma, Q. Gao, A. Dogan, M. Kizaki, M. Roshal, Y. Liu, and M. Yabe. 2023. Activating *STAT5B* mutations can cause both primary hypereosinophilia and lymphocyte-variant hypereosinophilia. Leuk. Lymphoma. 64:238–241. 10.1080/10428194.2022.2131413 36308018 PMC11026062

[64] Lamy, T., A. Moignet, and T.P. Loughran Jr. 2017. LGL leukemia: From pathogenesis to treatment. Blood. 129:1082–1094. 10.1182/blood-2016-08-692590 28115367

[65] Savasan, S., B. Al-Qanber, S. Buck, E. Wakeling, and M. Gadgeel. 2020. Clonal T-cell large granular lymphocyte proliferations in childhood and young adult immune dysregulation conditions. Pediatr. Blood Cancer. 67:e28231. 10.1002/pbc.28231 32124536

[66] Marchand, T., Pastoret, C., Moignet, A., Roussel, M., and Lamy, T. 2024. Large granular lymphocyte leukemia: A clonal disorder with autoimmune manifestations. Hematol. Am. Soc. Hematol. Educ. Program. 2024:143–149. 10.1182/hematology.2024000539 PMC11665628 39644019

[67] Barila, G., A. Grassi, H. Cheon, A. Teramo, G. Calabretto, J. Chahal, C. Vicenzetto, J. Almeida, B.C. Shemo, M. Shi, . 2023. Tγδ LGLL identifies a subset with more symptomatic disease: Analysis of an international cohort of 137 patients. Blood. 141:1036–1046. 10.1182/blood.2021013489 36096473 PMC10163282

[68] Bhattacharya, D., A. Teramo, V.R. Gasparini, J. Huuhtanen, D. Kim, J. Theodoropoulos, G. Schiavoni, G. Barilà, C. Vicenzetto, G. Calabretto, . 2022. Identification of novel STAT5B mutations and characterization of TCRβ signatures in CD4+ T-cell large granular lymphocyte leukemia. Blood Cancer J. 12:31. 10.1038/s41408-022-00630-8 35210405 PMC8873566

[69] Gray, J.I., D.P. Caron, S.B. Wells, R. Guyer, P. Szabo, D. Rainbow, C. Ergen, K. Rybkina, M.C. Bradley, R. Matsumoto, . 2024. Human gammadelta T cells in diverse tissues exhibit site-specific maturation dynamics across the life span. Sci. Immunol. 9:eadn3954. 10.1126/sciimmunol.adn3954 38848342 PMC11425769

[70] Arcila, M.E., W. Yu, M. Syed, H. Kim, L. Maciag, J. Yao, C. Ho, K. Petrova, C. Moung, P. Salazar, . 2019. Establishment of Immunoglobulin heavy (IGH) chain clonality testing by next-generation sequencing for routine characterization of B-cell and plasma cell neoplasms. J. Mol. Diagn. 21:330–342. 10.1016/j.jmoldx.2018.10.008 30590126 PMC6436112

[71] Keller, B., and K. Warnatz. 2023. T-be^thigh^CD21^low^ B cells: The need to unify our understanding of a distinct B cell population in health and disease. Curr. Opin. Immunol. 82:102300. 10.1016/j.coi.2023.102300 36931129

[72] Kobets, A.J., S. Ahmad, A. Boyke, D. Oriko, R. Holland, R. Eisenberg, S.A.N. Alavi, and R. Abbott. 2023. STAT5b gain-of-function disease in a child with mycobacterial osteomyelitis of the skull: Rare presentation of an emerging disease entity. Childs Nerv. Syst. 39:2071–2077. 10.1007/s00381-023-05997-y 37243811

[73] Schmitt, E.G., N. Saucier, S.I. Risma, S.N. Arbag, A. Kolicheski, A.J. Paul, T.L. Toler, K. Semkiu, S.S. Mar, J.D. Milner, . 2025. Mosaic STAT5B gain-of-function associated with demyelinating disease and autoimmunity. J. Hum. Immun. 1:e20250060. 10.70962/jhi.20250060 40874233 PMC12379004

[74] Cheon, H., J.C. Xing, K.B. Moosic, J. Ung, V.W. Chan, D.S. Chung, M.F. Toro, O. Elghawy, J.S. Wang, C.E. Hamele, . 2022. Genomic landscape of TCRαβ and TCRγδ T-large granular lymphocyte leukemia. Blood. 139:3058–3072. 10.1182/blood.2021013164 35015834 PMC9121841

[75] Morimoto, C., and S.F. Schlossman. 1998. The structure and function of CD26 in the T-cell immune response. Immunol. Rev. 161:55–70. 10.1111/j.1600-065x.1998.tb01571.x 9553764

[76] Van Acker, H.H., A. Capsomidis, E.L. Smits, and V.F. Van Tendeloo. 2017. CD56 in the immune system: More than a marker for cytotoxicity? Front. Immunol. 8:892. 10.3389/fimmu.2017.00892 28791027 PMC5522883

[77] Kuttruff, S., S. Koch, A. Kelp, G. Pawelec, H.-G. Rammensee, and A. Steinle. 2009. NKp80 defines and stimulates a reactive subset of CD8 T cells. Blood. 113:358–369. 10.1182/blood-2008-03-145615 18922855

[78] Brenchley, J.M., N.J. Karandikar, M.R. Betts, D.R. Ambrozak, B.J. Hill, L.E. Crotty, J.P. Casazza, J. Kuruppu, S.A. Migueles, M. Connors, . 2003. Expression of CD57 defines replicative senescence and antigen-induced apoptotic death of CD8+ T cells. Blood. 101:2711–2720. 10.1182/blood-2002-07-2103 12433688

[79] Palmer, B.E., N. Blyveis, A.P. Fontenot, and C.C. Wilson. 2005. Functional and phenotypic characterization of CD57+CD4+ T cells and their association with HIV-1-induced T cell dysfunction. J. Immunol. 175:8415–8423. 10.4049/jimmunol.175.12.8415 16339584

[80] Croker, B.A., H. Kiu, and S.E. Nicholson. 2008. SOCS regulation of the JAK/STAT signalling pathway. Semin. Cell Dev. Biol. 19:414–422. 10.1016/j.semcdb.2008.07.010 18708154 PMC2597703

[81] Zuberbuehler, M.K., M.E. Parker, J.D. Wheaton, J.R. Espinosa, H.R. Salzler, E. Park, and M. Ciofani. 2019. The transcription factor c-Maf is essential for the commitment of IL-17-producing γδ T cells. Nat. Immunol. 20:73–85. 10.1038/s41590-018-0274-0 30538336 PMC6294311

[82] Fu, Y., J. Wang, G. Panangipalli, B.J. Ulrich, B. Koh, C. Xu, R. Kharwadkar, X. Chu, Y. Wang, H. Gao, . 2020. STAT5 promotes accessibility and is required for BATF-mediated plasticity at the Il9 locus. Nat. Commun. 11:4882. 10.1038/s41467-020-18648-6 32985505 PMC7523001

[83] Yang, X.P., K. Ghoreschi, S.M. Steward-Tharp, J. Rodriguez-Canales, J. Zhu, J.R. Grainger, K. Hirahara, H.-W. Sun, L. Wei, G. Vahedi, . 2011. Opposing regulation of the locus encoding IL-17 through direct, reciprocal actions of STAT3 and STAT5. Nat. Immunol. 12:247–254. 10.1038/ni.1995 21278738 PMC3182404

[84] Liu, X., H. Lu, T. Chen, K.C. Nallaparaju, X. Yan, S. Tanaka, K. Ichiyama, X. Zhang, L. Zhang, X. Wen, . 2016. Genome-wide analysis identifies Bcl6-controlled regulatory networks during T follicular helper cell differentiation. Cell Rep. 14:1735–1747. 10.1016/j.celrep.2016.01.038 26876184 PMC4975778

[85] Rani, A., B. Afzali, A. Kelly, L. Tewolde-Berhan, M. Hackett, A.S. Kanhere, I. Pedroza-Pacheco, H. Bowen, S. Jurcevic, R.G. Jenner, . 2011. IL-2 regulates expression of C-MAF in human CD4 T cells. J. Immunol. 187:3721–3729. 10.4049/jimmunol.1002354 21876034 PMC3432901

[86] Canaria, D.A., B. Yan, M.G. Clare, Z. Zhang, G.A. Taylor, D.L. Boone, M. Kazemian, and M.R. Olson. 2021. STAT5 represses a STAT3-independent Th17-like program during Th9 cell differentiation. J. Immunol. 207:1265–1274. 10.4049/jimmunol.2100165 34348976 PMC8387395

[87] Kreisel, D., D. Sankaran, A.D. Wells, and L.A. Turka. 2002. Interleukin-2-mediated survival and proliferative signals are uncoupled in T lymphocytes that fail to divide after activation. Am. J. Transpl. 2:120–128. 10.1034/j.1600-6143.2002.020202.x 12099513

[88] Liao, W., J.X. Lin, and W.J. Leonard. 2011. IL-2 family cytokines: New insights into the complex roles of IL-2 as a broad regulator of T helper cell differentiation. Curr. Opin. Immunol. 23:598–604. 10.1016/j.coi.2011.08.003 21889323 PMC3405730

[89] Ai, W., H. Li, N. Song, L. Li, and H. Chen. 2013. Optimal method to stimulate cytokine production and its use in immunotoxicity assessment. Int. J. Environ. Res. Public Health. 10:3834–3842. 10.3390/ijerph10093834 23985769 PMC3799516

[90] Zhu, J., J. Cote-Sierra, L. Guo, and W.E. Paul. 2003. Stat5 activation plays a critical role in Th2 differentiation. Immunity. 19:739–748. 10.1016/s1074-7613(03)00292-9 14614860

[91] Rajala, H.L., K. Porkka, J.P. Maciejewski, T.P. Loughran Jr., and S. Mustjoki. 2014. Uncovering the pathogenesis of large granular lymphocytic leukemia-novel STAT3 and STAT5b mutations. Ann. Med. 46:114–122. 10.3109/07853890.2014.882105 24512550

[92] Neven, Q., C. Boulanger, A. Bruwier, M. de Ville de Goyet, I. Meyts, L. Moens, A. Van Damme, and B. Brichard. 2021. Clinical spectrum of Ras-associated autoimmune leukoproliferative disorder (RALD). J. Clin. Immunol. 41:51–58. 10.1007/s10875-020-00883-7 33011939

[93] Calvo, K.R., S. Price, R.C. Braylan, J.B. Oliveira, M. Lenardo, T.A. Fleisher, and V.K. Rao. 2015. JMML and RALD (Ras-associated autoimmune leukoproliferative disorder): Common genetic etiology yet clinically distinct entities. Blood. 125:2753–2758. 10.1182/blood-2014-11-567917 25691160 PMC4424627

[94] Steinway, S.N., F. LeBlanc, and T.P. Loughran Jr. 2014. The pathogenesis and treatment of large granular lymphocyte leukemia. Blood Rev. 28:87–94. 10.1016/j.blre.2014.02.001 24679833 PMC4155502

[95] Bravo-Perez, C., C. Gurnari, J. Huuhtanen, N. Kawashima, L. Guarnera, A. Mandala, N.D. Williams, C. Haddad, M. Witt, S. Unlu, . 2025. Inborn errors of immunity underlie clonal T cell expansions in large granular lymphocyte leukemia. J. Clin. Invest. 135:e184431. 10.1172/JCI184431 40309770 PMC12043085

[96] Loureiro, J.P., A. Vacchini, G. Berloffa, J. Devan, V. Schaefer, V. Nosi, R. Colombo, A. Beshirova, G. Montanelli, B. Meyer, . 2025. Recognition of MR1-antigen complexes by TCR Vγ9Vδ2. Front. Immunol. 16:1519128. 10.3389/fimmu.2025.1519128 40040716 PMC11876030

[97] Sagar & Ehl, S., and S. Ehl. 2025. γδ T cells and inborn errors of immunity. Eur. J. Immunol. 55:e51457. 10.1002/eji.202451457 40556329 PMC12188109

[98] Lepore, M., A. Kalinichenko, S. Calogero, P. Kumar, B. Paleja, M. Schmaler, V. Narang, F. Zolezzi, M. Poidinger, L. Mori, and G. De Libero. 2017. Functionally diverse human T cells recognize non-microbial antigens presented by MR1. Elife. 6:e24476. 10.7554/eLife.24476 28518056 PMC5459576

[99] Seitz, L., D. Gaitan, C.M. Berkemeier, C.T. Berger, and M. Recher. 2023. Cluster analysis of flowcytometric immunophenotyping with extended T cell subsets in suspected immunodeficiency. Immun. Inflamm. Dis. 11:e1106. 10.1002/iid3.1106 38156376 PMC10698832

[100] Glier, H., I. Heijnen, M. Hauwel, J. Dirks, S. Quarroz, T. Lehmann, A. Rovo, K. Arn, T. Matthes, C. Hogan, . 2019. Standardization of 8-color flow cytometry across different flow cytometer instruments: A feasibility study in clinical laboratories in Switzerland. J. Immunol. Methods. 475:112348. 10.1016/j.jim.2017.07.013 28760670

[101] Burgener, A.V., G.R. Bantug, B.J. Meyer, R. Higgins, A. Ghosh, O. Bignucolo, E.H. Ma, J. Loeliger, G. Unterstab, M. Geigges, . 2019. SDHA gain-of-function engages inflammatory mitochondrial retrograde signaling via KEAP1-Nrf2. Nat. Immunol. 20:1311–1321. 10.1038/s41590-019-0482-2 31527833

[102] Picard, C., H. Bobby Gaspar, W. Al-Herz, A. Bousfiha, J.-L. Casanova, T. Chatila, Y.J. Crow, C. Cunningham-Rundles, A. Etzioni, J.L. Franco, . 2018. International union of immunological societies: 2017 primary immunodeficiency diseases committee report on inborn errors of immunity. J. Clin. Immunol. 38:96–128. 10.1007/s10875-017-0464-9 29226302 PMC5742601

[103] Hahne, F., N. LeMeur, R.R. Brinkman, B. Ellis, P. Haaland, D. Sarkar, J. Spidlen, E. Strain, and R. Gentleman. 2009. flowCore: a Bioconductor package for high throughput flow cytometry. BMC Bioinformatics. 10:106. 10.1186/1471-2105-10-106 19358741 PMC2684747

[104] Levine, J.H., E.F. Simonds, S.C. Bendall, K.L. Davis, E.-aD. Amir, M.D. Tadmor, O. Litvin, H.G. Fienberg, A. Jager, E.R. Zunder, . 2015. Data-driven phenotypic dissection of AML reveals progenitor-like cells that correlate with prognosis. Cell. 162:184–197. 10.1016/j.cell.2015.05.047 26095251 PMC4508757

[105] Kolde, R. 2019. Pheatmap: Pretty heatmaps. R package version 1, 726.

[106] McInnes, L., J. Healy, and J. Melville. 2018. UMAP: Uniform manifold approximation and projection for dimension reduction. arXiv. 10.48550/arXiv.1802.03426 (Preprint posted September 18, 2020).

