# Supplementary material for: TCR γδ cell–specific STAT5 gain of function induces a druggable chronic human immune dysregulation
Source: J Hum Immun. 2026 Aug 10;2(5):e20250175. doi: 10.70962/jhi.20250175 (PMC13455652; doi:10.70962/jhi.20250175)

Supplemental Figure 1E

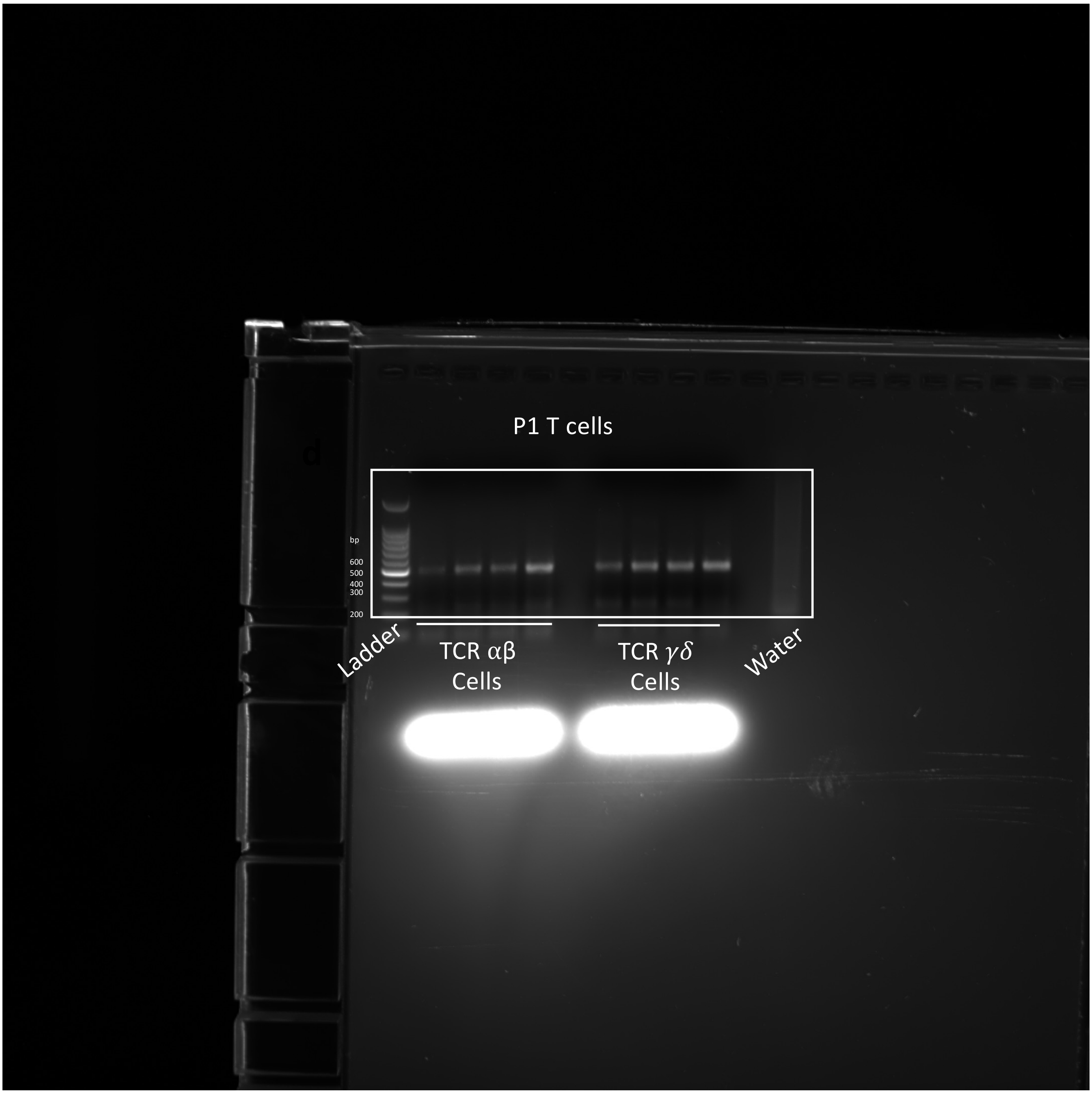

Supplemental Figure 1F

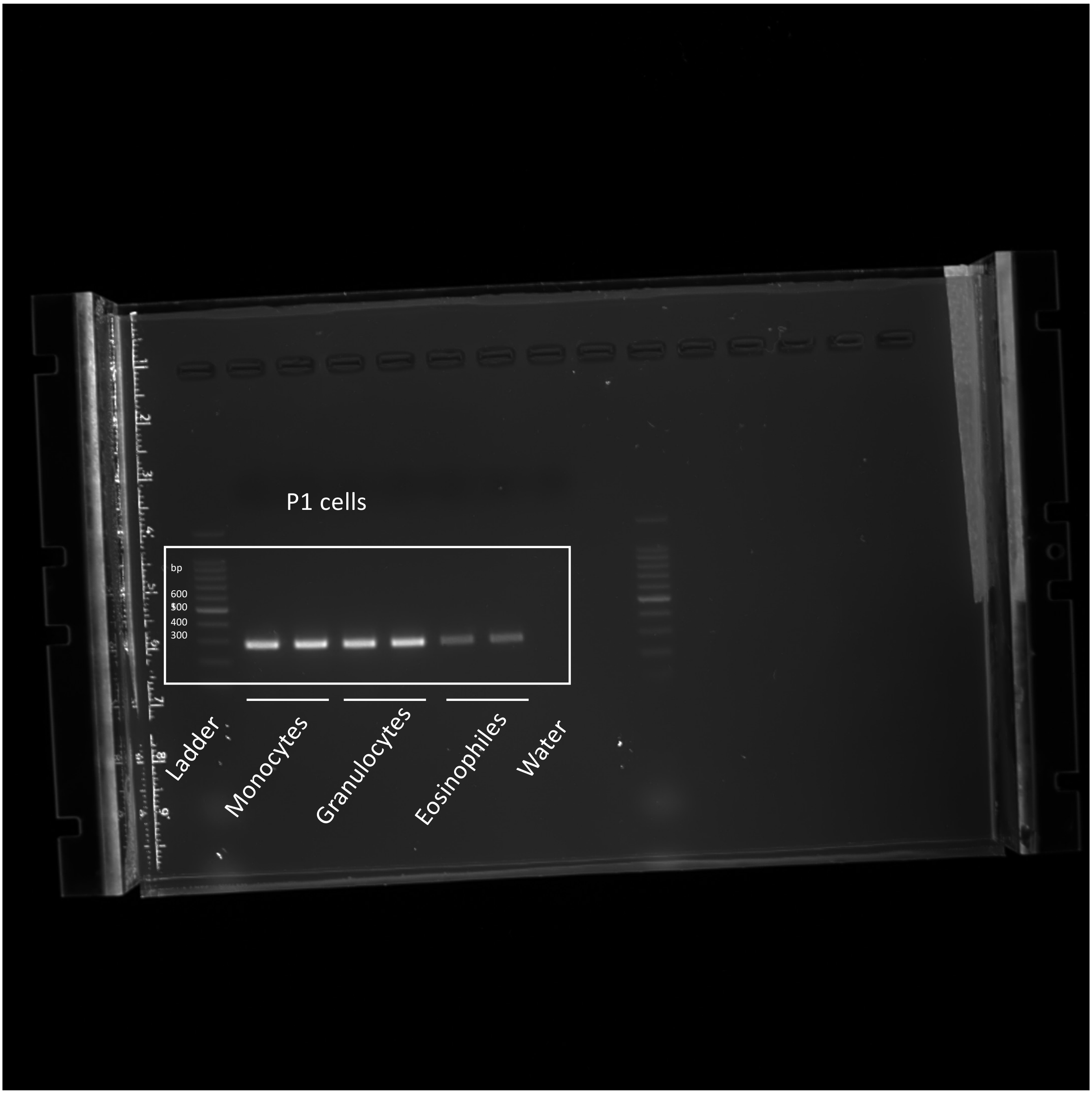

Supplement: Supplementary file 1 [file jhi_20250175_sourcedatafs1.pdf]
